# Recent Advances in the Synthesis and Antioxidant Activity of Low Molecular Mass Organoselenium Molecules

**DOI:** 10.3390/molecules28217349

**Published:** 2023-10-30

**Authors:** João M. Anghinoni, Paloma T. Birmann, Marcia J. da Rocha, Caroline S. Gomes, Michael J. Davies, César A. Brüning, Lucielli Savegnago, Eder J. Lenardão

**Affiliations:** 1 Laboratory of Clean Organic Synthesis (LASOL), Center of Chemical, Pharmaceutical and Food Sciences (CCQFA), Federal University of Pelotas (UFPel), P.O. Box 354, Pelotas 96010-900, RS, Brazil; joaomarcos9641@gmail.com (J.M.A.); carosigomes@gmail.com (C.S.G.); 2 Neurobiotechnology Research Group (GPN), Federal University of Pelotas (UFPel), P.O. Box 354, Pelotas 96010-900, RS, Brazil; paloma_birmann@hotmail.com; 3 Laboratory of Biochemistry and Molecular Neuropharmacology (LABIONEM), Center of Chemical, Pharmaceutical and Food Sciences (CCQFA), Federal University of Pelotas (UFPel), P.O. Box 354, Pelotas 96010-900, RS, Brazil; marciajr725@gmail.com; 4 Department of Biomedical Sciences, Panum Institute, University of Copenhagen, Building 12.6, Blegdamsvej 3, 2200 Copenhagen, Denmark; davies@sund.ku.dk

**Keywords:** antioxidant, organoselenium, GPx-like, DPPH, ABTS, FRAP, catalase, SOD, lipid peroxidation

## Abstract

Selenium is an essential trace element in living organisms, and is present in selenoenzymes with antioxidant activity, like glutathione peroxidase (GPx) and thioredoxin reductase (TrxR). The search for small selenium-containing molecules that mimic selenoenzymes is a strong field of research in organic and medicinal chemistry. In this review, we review the synthesis and bioassays of new and known organoselenium compounds with antioxidant activity, covering the last five years. A detailed description of the synthetic procedures and the performed in vitro and in vivo bioassays is presented, highlighting the most active compounds in each series.

## 1. Introduction

Selenium is an essential trace element in living organisms, being incorporated in the 21st amino acid selenocysteine (Sec), which is present in the selenoenzymes with antioxidant activity including multiple isoforms of glutathione peroxidase (GPx), thioredoxin reductase (TrxR), and other selenoproteins [1,2]. The incorporation of selenium into different molecules permits the synthesis of many *Se*-containing compounds to explore their biochemistry and potential antioxidant properties [3,4,5]. The protective properties of many different classes of organoselenium compounds, including diselenides, *N*- or *Se*-heterocycles, selenides, and Ebselen derivatives have been described [6,7,8,9,10]. This review highlights new research on the synthesis and antioxidant properties of low-molecular mass organoselenium compounds over the period 2018 to early 2023. To prepare this systematic review, we have examined articles, reviews, and book chapters using the databases SciFinder, Web of Science, Scopus, PubMed, and Google Scholar. The search terms used were “antioxidant”, “organoselenium”, “GPx-like”, “selenides”, “glutathione peroxidase”, and “selenium”.

**Figure 1:**
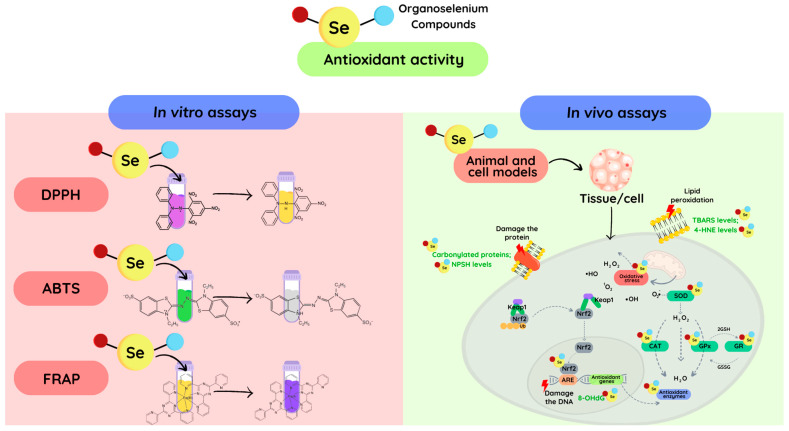
The routes of the mainly antioxidant assays for exploring the antioxidant properties of novel organoselenium compounds. The antioxidant properties can be studied in a cell through the reduction of the oxidative damage caused to DNA, proteins, and cell membranes, or by mimicking antioxidant enzymes (SOD, CAT, GPx, etc.). Additionally, the redox activity can be observed in vitro through the reduction of oxidant concentrations or synthetic radicals, such as DPPH and ABTS. Finally, oxidants can be directly measured using specific techniques, such as electron paramagnetic (spin) resonance and fluorescent probes. In the DPPH assay, after the reduction of the DPPH radical the color changes from purple to pale yellow. In the ABTS assay, upon reduction of the ABTS radical, the green color discoloration occurs. In the FRAP test, the reduction of Fe^3+^ yields a violet-blue color.

Multiple different methods have been used to investigate and quantify the antioxidant properties of organoselenium compounds that include in vitro assays involving hydrogen atom or electron transfer. These include approaches that measure the extent of damage to biologically important targets such as lipids, proteins, and DNA via the quantification of their oxidation products. This can be achieved via assays that measure loss of the parent species or formation of generic or specific oxidation products. Examples of the former include total hydroperoxides and alcohols, and lipid or protein carbonyls. Examples of specific oxidation products include isoprostanes and regio- and stereoisomeric lipid and cholesterol hydroperoxides and alcohols, oxidized DNA bases, and species formed on individual amino acid sidechains (carbonyls, alcohols, oxyacids, sulfoxides, chlorinated, and nitrated species) [11,12,13]. The overall extent of oxidation in complex systems can also be examined by measuring oxygen consumption via assays such as the total oxygen radical absorbance capacity (ORAC). Other in vitro methods are based on electron transfer, for example, ferric iron-reducing antioxidant power (FRAP), 2,2-diphenyl-1-picrylhydrazyl (DPPH), and 2,2′-azinobis [3-ethylbenzothiazoline-6-sulfonic acid] (ABTS) [14,15,16], though these assays cannot be readily applied to complex systems and the data obtained from such assays often show poor correlation with biological assays. Thus, techniques that can be applied to cells or animal models are the most important and reproducible with regard to assessing biological activity. A larger number of studies have reported changes in the activity or protein levels of enzymes such as superoxide dismutase (SOD), catalase (CAT), and GPxs, and changes in gene expression [15]. However, it needs to recognize that enzyme levels and activities can be altered by many pathways; therefore, such data are not direct measures of antioxidant activity (Figure 1). Direct measurement of oxidants, and particularly reactive radicals, is notoriously difficult in complex systems due to the low steady state concentration of such species. Valuable data have been obtained using highly specific techniques such as electron paramagnetic (spin) resonance (EPR or ESR); however, even with this method, trapping agents (spin traps) or spin probes need to be employed for highly reactive species, and these agents have considerable drawbacks and caveats [17]. Fluorescent probes (e.g., dichloro-dihydrofluorescein diacetate, DCFH-DA; dihydroethidium, DHE) have also been widely employed to detect oxidant generation, but many of these probes suffer from considerable problems and artefacts; therefore, large numbers of control experiments are required, and the resulting data need to be treated with great care [12,18].

This review is divided in two main parts: firstly, a short introduction to oxidative stress is provided; secondly, the synthesis of organoselenium compounds and their potential protective activities is discussed at length. This second part is organized according to the class of organoselenium compounds: diselenides, Ebselen derivatives, *Se*-functionalized heterocycles, *Se*-heterocycles, selenides, and miscellaneous.

## 2. Oxidative Stress

Reactive oxidants/species (henceforth, RS), including reactive oxygen species (often termed ‘ROS’, though this is a general term for a large group of species with very different reactivities and effects) are produced both deliberately and accidently during oxygen metabolism. These species can have beneficial effects by mediating cell signaling and harming or killing invading pathogens when they are generated inappropriately. Major ‘ROS’ species include hydrogen peroxide (H_2_O_2_), other peroxides (ROOH), the superoxide radical anion (O_2_^•−^), hydroxyl radical (HO^•^), singlet oxygen (^1^O_2_), peroxyl radicals (ROO^•^), and alkoxyl radicals (RO^•^). The intracellular concentration of oxidants is carefully regulated through a delicate interplay between their production and removal mechanisms, which involves a complex and diverse mixture of antioxidant defenses. Under normal physiological conditions, oxidants play a crucial role in regulating signaling pathways that control numerous cellular processes (‘oxidative eustress’). However, excessive oxidant levels can lead to cell and tissue damage (‘oxidative distress’) [19,20].

Antioxidants mitigate potential detrimental effects of RS, including ROS and related reactive nitrogen, carbon, sulfur, and chlorine species. If oxidant production overwhelms the capacity of antioxidants to eliminate them, or if antioxidant production is compromised, oxidative stress (‘distress’) occurs. Enzymes play a critical role in protecting cells, the extracellular environment and tissues/organs against damage caused by RS [21,22]. These enzymes, which both prevent and repair damage, are supplemented by a battery of low-molecular mass species that can directly scavenge RS, though these are often less important than enzymatic systems.

The superoxide dismutase (SOD) family is a key protective system which catalyzes the conversion of O_2_^•−^ into H_2_O_2_ and molecular oxygen (O_2_). The resulting H_2_O_2_ is then neutralized by multiple enzyme systems including multiple peroxiredoxin (Prxs) and glutathione peroxidase (GPx) isoforms, as well as catalase (CAT). Catalase converts H_2_O_2_ into water (H_2_O) and oxygen (O_2_) without the use of co-factors, whereas the activities of Prxs and GPxs reduce H_2_O_2_ (and lipid hydroperoxides in the case of GPx4) to H_2_O (or lipid alcohols for GPx4) at the expense of thiol-dependent co-factors. The activity of Prxs is maintained by the thioredoxin (Trx)/thioredoxin reductase (TrxR)/NAPDH system, whereas GPxs use glutathione (GSH) as a cofactor. The glutathione disulfide (GSSG) generated from GSH oxidation is reduced by glutathione reductase using NADPH as the reducing co-factor [23,24]. The glutathione *S*-transferase (GST) and glutaredoxin families are also involved in protection against oxidative stress. The GST isoforms catalyze the adduction of GSH to a variety of toxic compounds and drugs, facilitating their elimination from the body [23,24,25]. Both the Prx and GPx systems are ultimately dependent on selenium, as both TxrRs and GPxs contain Sec residues in their active sites.

Collectively, these antioxidant enzymes act as an efficient defense system, controlling oxidant levels within and external to cells, and safeguard biological systems against oxidative damage [25]. However, imbalances between oxidant production and their removal can lead to significant detrimental effects to various cellular components, and hence health problems, including neurodegenerative, cardiovascular, lung, skin, kidney and liver diseases, and psychiatric disorders [26,27,28]. Adequate selenium levels are therefore critical to the maintenance of cellular antioxidant levels, and supplementation with biologically-available Se species may be beneficial, particularly in the case of deficiency [29,30,31,32].

**Figure 2:**
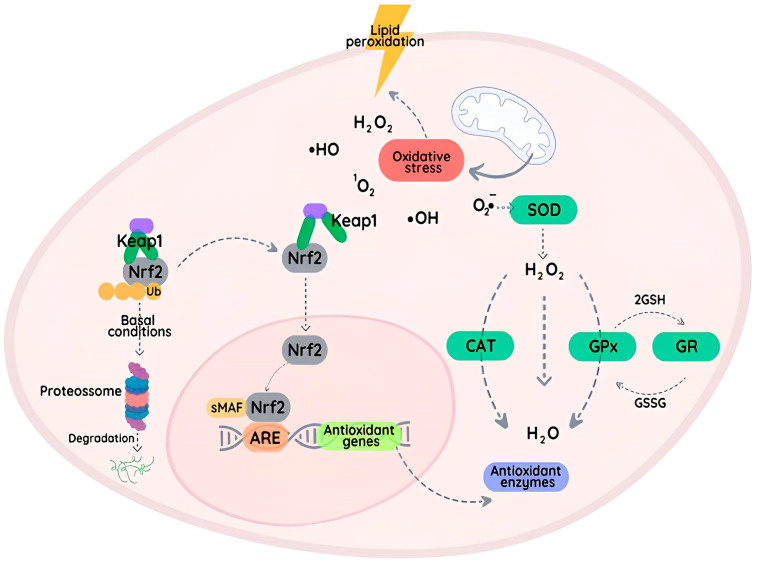
The Nrf2 pathway is regulated by interactions between Nrf2 and the Keap1 protein in the cytoplasm. Under basal conditions, Nrf2 expression is maintained at low levels through proteasome activity. When the level of reactive species is elevated (oxidative stress), Keap1 is modified, resulting in the accumulation and translocation of Nrf2 to the nucleus, where it binds to AREs and activates the transcription of antioxidant genes, including those coding for SOD, CAT, and GPxs. This adaptive response plays a critical role in protecting cells against damage.

The Nrf2 (nuclear factor erythroid 2-related factor 2) pathway is an important cellular transcription factor involved in cellular antioxidant responses [33], as it regulates the expression of genes responsible for synthesizing antioxidant enzymes [33,34]. In the basal state, the Nrf2 is present in the cytoplasm and is associated with the Keap1 protein. This protein targets Nrf2 for rapid proteasomal degradation and thereby prevents its accumulation and translocation to the nucleus. However, Keap1 contains a number of highly reactive cysteine residues and modification of these can result in decreased Keap1 activity and an accumulation of newly synthesized Nrf2 in both the cytosol and nucleus (Figure 2) [33,34]. Nuclear Nrf2 forms a heterodimer with Maf transcription factor proteins and binds to antioxidant response elements (AREs) located in the promoters of target genes, thereby promoting their transcription. These genes include those coding for SOD, CAT, and GPx [33]. In addition, Nrf2 also regulates the expression of genes involved in the synthesis and regeneration of GSH [33,34,35]. GSH therefore plays a central role in protecting against oxidative damage by acting as a reducing enzyme cofactor, as a direct scavenger of oxidants, and also as an agent adducted to exogenous chemicals to enhance their excretion.

Oxidants also regulate a number of other stress response pathways involving nuclear factor-κ light chain-enhancer of activated B cells (NF-κB), hypoxia-inducible factor (HIF), oestrogen-related receptor (ERR), forkhead box O transcription factor (FOXOs) peroxisome proliferator-activated receptor-γ co-activator 1α (PPAR), peroxisome proliferator-activated receptor-γ co-activator 1α (PGC1a), cellular tumour antigen p53 (p53), 5-adenosine monophosphate-activated protein kinase (AMPK), mammalian target of rapamycin (mTOR), glyceraldehyde-3-phosphate dehydrogenase, (GAPDH), sirtuin protein family (SIRT), and uncoupling protein 1 (UCP) [18,36].

These data indicate a key role for both thiols and Se species in maintaining the correct redox state within cells and tissues and has given rise to widespread interest in the maintenance or enhancement of these defense systems, particularly in pathologies or cases of genetic or nutritional deficiency, where these systems may be compromised. This has resulted in considerable efforts to design and test low molecular mass Se species that can mimic the activity of these key selenium and sulfur dependent systems.

## 3. Synthesis and Antioxidant Evaluation of Organoselenium Compounds

### 3.1. Diselenides

**Scheme 1:**
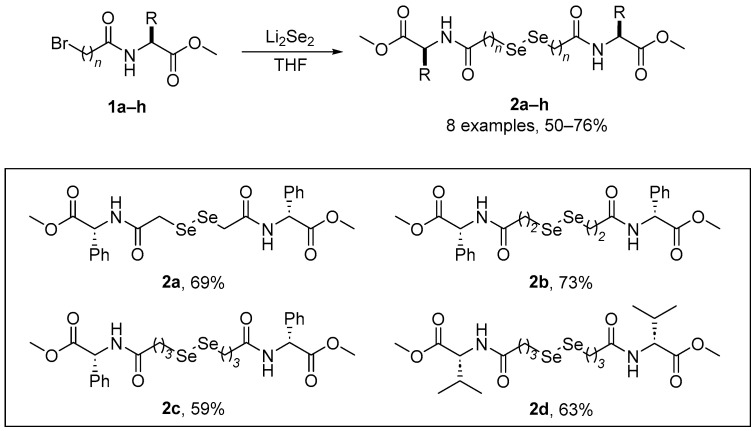
Synthesis of diselenoamino acid derivatives **2**.

As part of the growing interest in chalcogen-containing amino acid derivatives as chiral building blocks in organic synthesis or for biological protection, Braga and co-workers [37] have reported a facile and inexpensive route for the preparation of a series of *Se*-substituted amino acids. A major advantage of this strategy is its modular construction, where modifications in the product structure can be easily introduced. The synthesis of the diselenides **2a**–**h** (Scheme 1) involves the reaction of bromo amides **1a**–**h** with Li_2_Se_2_ in THF, affording good yields of the expected products (50–76%). The yields of the diselenides derived from *L*-phenylalanine were not affected by the increase in the carbon chain length. These diselenoamino acid derivatives has been evaluated for GPx- and TxrR-like activity, thiol oxidase, and thiobarbituric acid reactive substances (TBARS, a commonly used, but rather indiscriminate assay for reactive carbonyls including malondialdehyde, MDA). Diselenide **2d**, derived from *L*-valine, showed the best GPx-like activity, followed by **2c** and **2b**. Compound **2a** was the least effective catalyst in the thiol-mediated reduction of H_2_O_2_. Compound **2d** presented the highest thiol oxidase activity, followed by compounds **2c** and **2b**. Compound **2a** was the less efficient oxidant of GSH (monothiol) and DTT (dithiol). The reactivity of diselenoamino acid derivatives with rat hepatic TrxR was tested:the order of reactivity with 5 μM of the diselenoamino acid derivatives was **2d** > **2c** > **2b** > **2a**. At 15 μM, compounds **2d** and **2c** increased the oxidation of NADPH from 7.5% to 11%, and from 6% to 9%, respectively, but compounds **2a** and **2b** oxidized less NADPH than observed at 5 μM, indicating that at 15 μM, they were probably inhibiting TrxR activity. In the TBARS assay, compounds **2d** and **2c** exhibited only a weak antioxidant activity against iron-induced lipid peroxidation, and compounds **2a** and **2b** were ineffective.

**Scheme 2:**
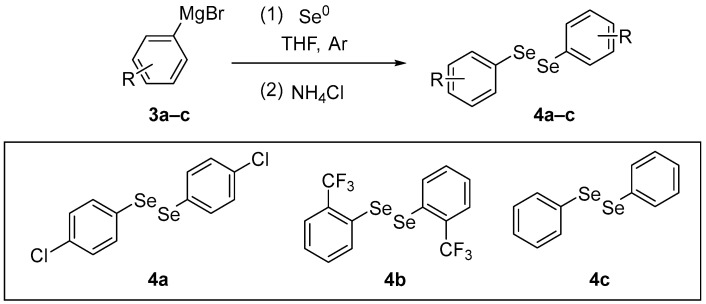
Synthesis of diselenides **4**.

4,4′-Dichlorodiphenyl diselenide (*p*-ClPhSe)_2_ **4a** is an organoselenium compound reported to have antioxidant, antidepressant-like, and neuroprotective actions. Nogueira and co-workers [38] have investigated whether the antioxidant activity and modulation of the glutamatergic system contribute to the antidepressant-like effect of (*p*-ClPhSe)_2_ in mice sub-chronically exposed to dexamethasone (DEX). 4,4′-Dichlorodiphenyl diselenide **4a** was synthesized according to the literature [39] through the reaction between the Grignard of *p*-chlorobromobenzene **3a** and elemental selenium in THF. After the formation of the selenolate intermediate, the reaction was quenched with saturated NH_4_Cl, and the resulting selenol was oxidized with atmospheric O_2_ to give the diselenide **4a** (Scheme 2). It was shown that (*p*-ClPhSe)_2_ has antioxidant actions in the prefrontal cortex of mice exposed to DEX. The animals received DEX (2 mg/kg, intraperitoneal [i.p.]) for 21 days. Then, the animals were treated with (*p*-ClPhSe)_2_ (1, 5, or 10 mg/kg, intragastrically [i.g.]) for 7 days. After the last treatment, the prefrontal cortex was removed to determine the levels of oxidation and CAT and SOD activities. At the tested doses, (*p*-ClPhSe)_2_ reduced oxidant levels and increased CAT activity, while at the highest dose (10 mg), it was effective against the decrease in SOD activity induced by DEX exposure.

Brüning et al. [40] have studied the effects of bis(*m*-(trifluoromethyl)phenyl) diselenide **4b** in a reserpine-induced pain-depression dyad model. Diselenide **4b** was prepared, as described in Scheme 2, using the Grignard Reagent obtained from 1-bromo-3-(trifluoromethyl)benzene. Mice were injected with reserpine 0.5 mg/kg, i.p. for three consecutive days, once a day, and then they received **4b** (10 mg/kg) for the next two days, once a day. Thirty minutes after the last administration of **4b**, the behavioral tests were carried out. Diselenide **4b** reversed the reserpine-increased thermal hyperalgesia and depressive-like behavior of mice. These effects appear to be related to modulation of oxidative stress, since **4b** normalized TBARS and 4-hydroxynonenal- (4-HNE) modified protein levels, markers of lipid oxidation increased by reserpine.

Dos Santos et al. [41] have reported a transcriptional approach to the redox and insulin-signaling pathway using zebrafish (*Danio rerio*) as a model organism. The authors investigated biomarkers underlying the protective effects of diphenyl diselenide **4c** against hyperglycemia. Zebrafish were fed a diet containing diphenyl diselenide (3 mg/kg) for 74 days. During the last 14 days of the study, they were exposed to a 111 mM glucose solution to induce a hyperglycemic state. Following this, the fish were euthanized. The brain tissue was analyzed for levels of TBARS and carbonylated proteins, non-protein sulfhydryl (NPSH) levels, as well as the activities of CAT, SOD, GPx, and GST and mRNA levels of FoxO3A, FoxO3B, Nrf2, GPx3A, SOD1, and SOD2. Compound **4c** counteracted the effects of hyperglycemia toward oxidation of lipids and proteins and elevated per se the brain levels of NPSH. In addition, **4c** attenuated the effect of hyperglycemia on SOD and increased the activity of GPx and GST enzymes. This compound also normalized the transcriptional levels of FoxO3A, FoxO3B, Nrf2, GPx3A, SOD1, and SOD2, suggesting that the antioxidant effect of **4c** in this model is associated with the regulation of oxidative stress.

In order to further examine the effects of Se compounds on hyperglycemia and its consequences, Shen and co-workers [42] have evaluated the preventive and therapeutic effects of **4c** against diabetic peripheral neuropathy (DPN), a common microvascular complication of both type 1 and type 2 diabetes mellitus. In vitro, the RSC96 cells were exposed to a very high glucose concentration (100 mM, much higher than detected in poorly controlled diabetes) and then treated with different concentrations of **4c** at 1, 10, 25, and 50 μM. These treatments decreased oxidant and malondialdehyde (MDA, a generic product of lipid and sugar oxidation) levels. This compound also activated Nrf2 signaling and inhibited Keap1 expression. In vivo, male rats received a single i.p. injection of streptozotocin (60 mg/kg). After five weeks, the animals were treated with **4c** (5 and 15 mg/kg, i.g); 12 weeks later, the rats were sacrificed, and the sciatic nerves removed for evaluation. Treatment with **4c** decreased MDA levels and increased GSH, SOD, CAT, and GPx levels in serum and sciatic nerves, reduced the level of Keap1, and stimulated Nrf2 signaling. Taken together, these data indicate that **4c** ameliorates DPN in rats by both ameliorating oxidant levels and by activating the Nrf2/Keap1 signaling pathway.

**Scheme 3:**
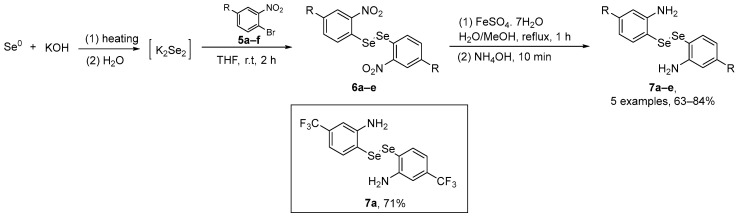
Synthesis of aniline-derived diselenides **7**.

Braga and co-workers [43] have described a simple and efficient route to access aniline-derived diselenides and evaluated their antioxidant/GPx-mimetic properties. The synthesis starts with the aromatic nucleophilic substitution of *o*-bromonitrobenzene **5a** with K_2_Se_2_ (generated in situ), affording bis(*o*-nitrobenzene) diselenide **6**. The reduction of bis(*o*-nitrobenzene) diselenides **6** by a well-established procedure using low-cost iron sulfate heptahydrate (FeSO_4_.7H_2_O) afforded the aniline-derivatives **7** (Scheme 3). The aniline-derived diselenides were evaluated GPx-like activity and in kinetic assays. Diselenide **7a**, substituted with the CF_3_ group, showed the best results, being five and two times more effective as a GPx mimetic than Ebselen and **4c**, respectively.

Studies have also been carried out to assess the effects of Se compounds against hyperglycemia-induced kidney damage (diabetic nephropathy). Thus, Zhou and co-workers [44] have examined the effects of **4c** supplementation on diabetic nephropathy in streptozotocin-treated rats. Male rats were subjected to i.p. injection of streptozotocin (60 mg/kg) following fasting for 10 h. After four weeks, they received **4c** once daily at 5 or 15 mg/kg for 12 weeks. The animals were then sacrificed, and blood and kidneys were collected for analysis. Administration of **4c** modulated GSH levels, the activities of GPx and SOD, and reduced MDA levels in serum and the kidneys. In addition, the compound regulated renal gene expression of Nrf2 and Nrf2-targeted antioxidant enzymes. These data indicate that **4c** has a renoprotective effect, which may be attributed to its ability to reduce oxidative stress caused by hyperglycemia. This effect may arise through the activation of the Nrf2/Keap1 system.

**Scheme 4:**
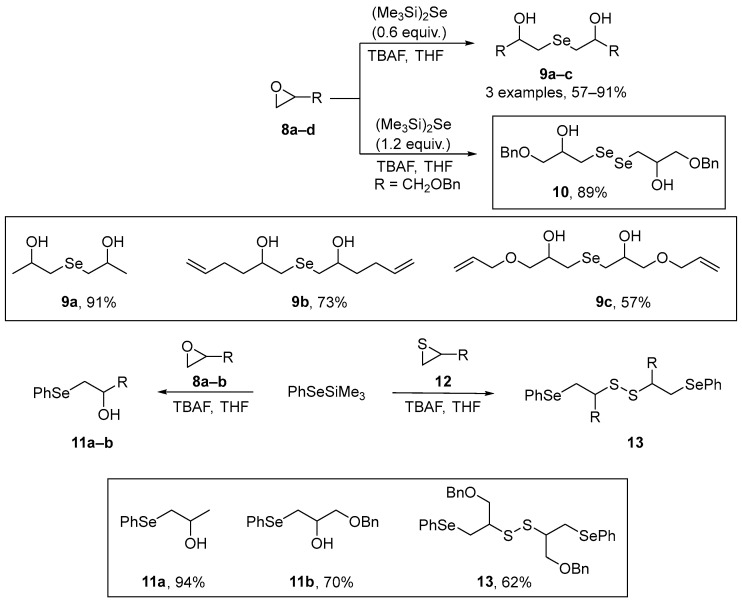
Synthesis of differently substituted β-hydroxy dialkyl- and alkyl-aryl selenides.

A number of substituted β-hydroxy- and β-amino dialkyl and alkyl-aryl chalcogenides have been synthesized by Capperucci et al. [45] through the ring-opening of epoxides and aziridines by selenium- or tellurium-centered nucleophiles. Synthesis of the symmetrical β-hydroxy selenides **9** and diselenide **10** was achieved through TBAF-induced ring-opening of the epoxides with (Me_3_Si)_2_Se [bis(trimethylsilyl)selenide or HMDSS]. By altering the reaction stoichiometry selective formation of **9** or **10** could be achieved (Scheme 4). In a related reaction, a fluoride ion-induced silicon-mediated procedure was used to generate the β-phenylseleno- alcohols **11** and disulfides **13** from (phenylseleno)trimethylsilane and the relevant epoxides or thiiranes. The antioxidant properties of the compounds were investigated in human dermal fibroblasts using the spectrofluorimetric DCFH assay. The cells were exposed to the compounds at 5–50 nM, and the buildup of fluorescence monitored. The results obtained revealed a significant antioxidant potential of these compounds. Thiol-peroxidase assays, using dithiothreitol (DTT), demonstrated that the compounds were effective catalysts of DTT oxidation.

**Scheme 5:**
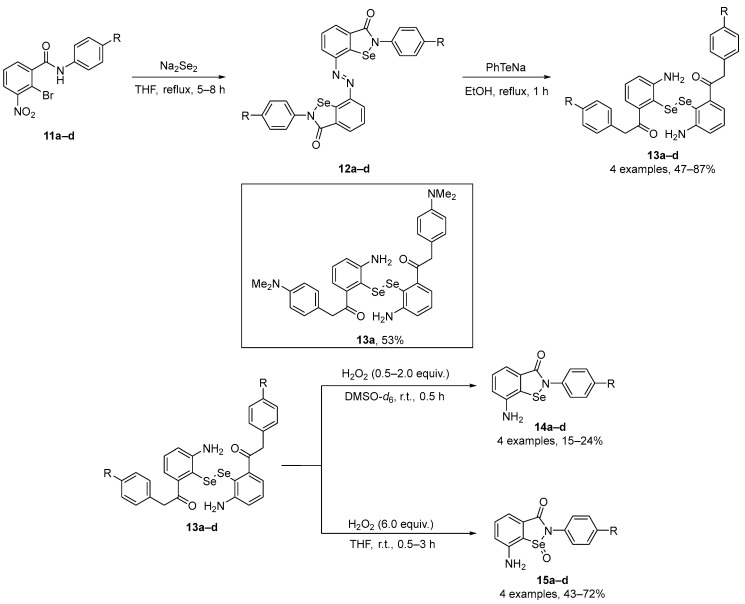
Synthesis of novel regenerable and multifunctional selenazolonamines **14** and **15**.

Singh and co-workers [46] have described the synthesis of novel regenerable and multifunctional selenazolonamines **14a**–**d** incorporating free -NH_2_ group near to the Se atom with remarkable protective and radical-trapping properties. The cyclic selenazolonamines **14a**–**d** were obtained by oxidation of the corresponding diselenides **13a**–**d** with H_2_O_2_ under controlled reaction conditions. Azo-bis-ebselen precursors **12a**–**d** were prepared by treating **11a**–**d** with Na_2_Se_2_ in dry THF under reflux, according to previously described by the authors [47]. In the sequence, diselenides **13a**–**d** were further utilized for synthesizing selenazolonamines **14a**–**d**. The reaction of **13a**–**d** with H_2_O_2_ and DMSO-d6 gave the products **14a**–**d** in 15–24% yield. To generate the corresponding selenoxides, the diselenides **13a**–**d** were treated with H_2_O_2_ in THF, giving **15a**–**d** in good yields (43–72%) (Scheme 5). Inhibition rates of conjugated diene (a generic lipid oxidation product) formation (R_inh_) and inhibition times (T_inh_) with and without ascorbate (AscOH) were evaluated for **14a**–**d** using linoleic acid as the oxidizable lipid. All the compounds at 40 μM diminished the extent of lipid peroxidation. GPx mimetic activity was also evaluated for the same compounds and diselenide **13a**, and the resulting data demonstrate that **14a**–**d** (20 μM), **15a**–**d** (20 μM), and **13a** (20 μM) have greater GPx-like activity than Ebselen. These new compounds therefore appear to be excellent mimics of GPxs and lipid-soluble antioxidants, protecting lipid membranes and mitochondrial DNA against oxidative stress caused by reactive species in cells.

**Scheme 6:**
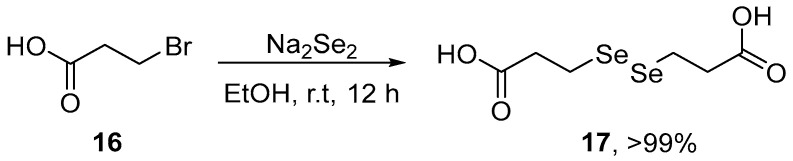
Synthesis of 3,3′-diselenodipropionic acid **17** (DSePA).

Deshmukh et al. [7] have reported data on the interaction of 3,3′-diselenodipropionic acid (DSePA) **17**, a well-known pharmacologically active diselenide, with HgCl_2_ and its ability to prevent HgCl_2_-induced toxicity in experimental cellular and mice models. DSePA was synthesized starting from the reaction of Na_2_Se_2_, generated in situ by the reaction of an ethanolic suspension of Se^0^ in the presence of NaBH_4_, with 3-bromopropanoic acid **16**. The resulting mixture was stirred for 12 h at room temperature to afford the expected diselenide in almost quantitative yield (Scheme 6). Pre-treatment with DSePA prevented HgCl_2_-induced oxidative stress in Chinese Hamster Ovary (CHO) cells, and significantly increased the activities of GPx and TrxR and the ratio GSH/GSSG. In vivo studies indicate that the administration of DSePA (2 mg/kg i.p.) prevented HgCl_2_-induced oxidative stress in mice. Pre-administration of DSePA significantly increased the levels of GPx, TrxR, and GSH/GSSH ratio and decreased TBARS levels in the liver and kidney of mice. Taken together, these results demonstrated that DSePA affords protection against Hg-induced oxidative stress in cell and animal models.

The kinetics of reaction of DSePA and a range of related species, with hypochlorous acid (HOCl), singlet oxygen (^1^O_2_), and a range of peroxides have been reported [48]. These data were compared with the corresponding sulfur-containing species and shown to be significantly higher. The data indicate considerable variations in the rates of reaction of selenols, selenides and diselenides with different oxidants, with the reactivity decreasing along the order selenols > selenides > diselenides. These data complement earlier kinetic and product data reported for sugar-derived selenides (see also below) with different oxidants [49,50,51].

Shen et al. [52] have investigated the capacity of diphenyl diselenide **4c** to alleviate tert-butyl hydrogen peroxide (TBHP)-induced oxidative stress, and in lipopolysaccharide (LPS) -induced inflammation in rat glomerular mesangial (HBZY-1) cells, and its potential as a candidate for the prevention and treatment of diabetic nephropathy. The antioxidant activity in HBZY-1 cells was determined by the production of intracellular oxidants and the levels of MDA, GSH, and SOD activity. The cells were exposed to TBHP (400 μM) with **4c** at different concentrations (10, 25, or 50 μM) for 24 h. After treatment, the cells were exposed to DCFH-DA and the fluorescence intensity was determined. Oxidation levels in TBHP-stimulated HBZY-1 cells were significantly decreased after treatment with **4c** at all concentrations. To evaluate the levels of MDA, GSH, and SOD activity, the HBZY-1 cells were exposed to TBHP (400 μM) and **4c** (10, 25, or 50 μM) for 24 h. Such treatment decreased MDA levels and increased intracellular GSH content and SOD activity in the TBHP-stimulated HBZY-1 cells. To address the antioxidant mechanism of **4c**, its effects on the protein expression of Nrf2 and downstream antioxidant enzymes were investigated by immunoblotting. Treatment with **4c** increased expression of Nrf2, NQO1, HO-1, and GCLC protein, and reduced expression of Keap1 in TBHP-treated HZBY-1 cells. Thus, activation of the Nrf2/Keap1 signaling pathway may be involved in the antioxidant mechanism of **4c**. It should be noted that the measurement of intracellular oxidants in these studies may have been compromised by the use of TBHP as the inducer of oxidative stress, as this is itself a powerful cell-penetrating oxidant.

**Scheme 7:**
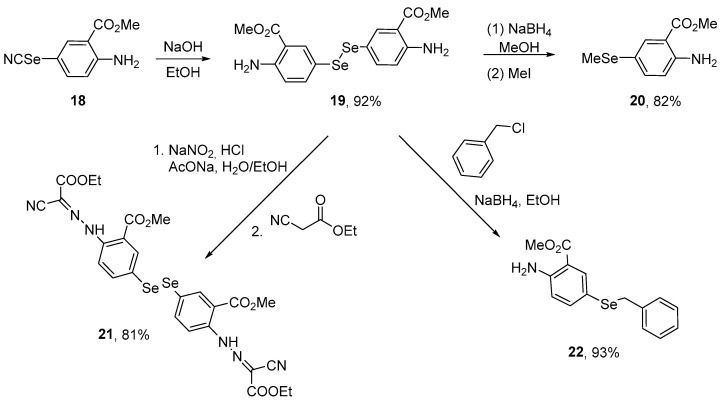
Synthesis of methyl anthranilate-based organodiselenide hybrids.

Incorporation of the diselenide functionality into the backbone of anthranilic acid has provided novel compounds designed to interfere with biotargets. Al Abdallah et al. [53] synthesized methyl anthranilate-based hybrid diselenides and evaluated their antioxidant activity using in vitro ABTS and DPPH assays. The selenocyanate **18** was submitted to hydrolysis using NaOH in EtOH to afford the diselenide **19** in 92% yield. Diselenide **19** was converted to selenide **20** (82% yield) through a one-pot alkaline reduction employing a 1:1 mixture of NaBH_4_ and NaOH in MeOH, followed by the reaction with methyl iodide (Scheme 7). The tested compounds exhibited antioxidant activity in both tests. Compounds **20**, **19**, **22**, **21**, and **18** exhibited 96%, 92%, 89%, 85%, and 81% of scavenging activities in the ABTS assay, respectively. In the DPPH assay, compounds **20**, **22**, **19**, **18**, and **21** exhibited 91%, 88%, 86%, 73%, and 69% scavenging activities, respectively. Whether such activity also occurs in more complex systems remains to be established.

### 3.2. Ebselen Derivatives

**Scheme 8:**
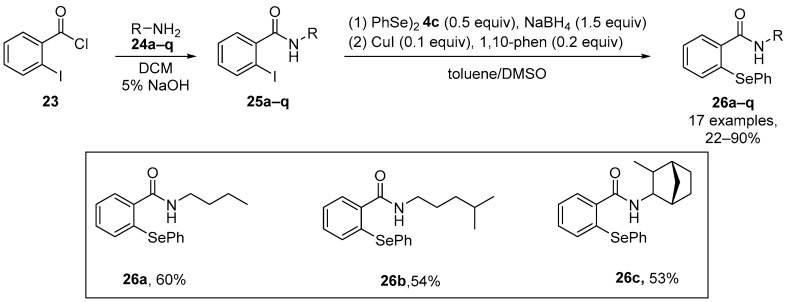
Synthesis of *N*-substituted unsymmetrical phenylselenides **26**.

Ebselen and analogues have been widely studied as redox modulators, and consequently Scianowski et al. [54] have synthesized novel Se-based species related to the ebselen skeleton. *N*-substituted unsymmetrical phenyl selenides containing an *o*-amido function were generated via copper-catalyzed nucleophilic substitution by PhSe**^-^** formed in situ from reduction of diphenyl diselenide by NaBH_4_. The rate of H_2_O_2_ removal by these compounds was evaluated indirectly via an indirect antioxidant assay using dithiothreitol (DTT) as a reducing thiol cofactor. The most potent antioxidants within this new series were the *N*-butyl-(**26a**), *N*-3-methylbutyl-(**26b**) and phenyl selenides (**26c**) (Scheme 8).

**Scheme 9:**
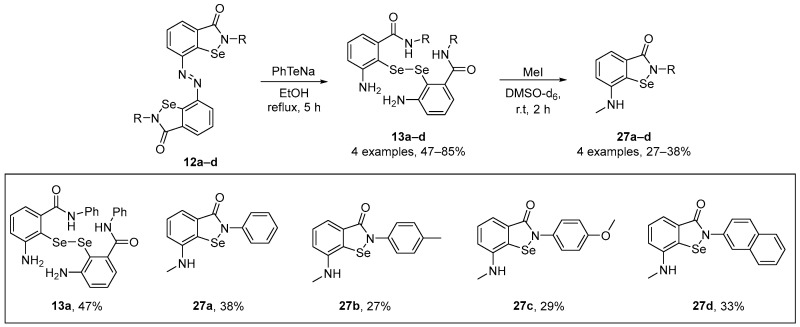
Synthesis of novel *N*-methylated ebselenamine derivatives **27**.

Kumar et al. [55] have described a straightforward synthesis of novel *N*-methylated ebselenamines and the capacity of these compounds to quench peroxyl radicals. These species showed higher activity than α-tocopherol and were regenerable in the presence of ascorbic acid. Such data contribute to a better understanding of ebselenamine antioxidants as potential drugs against oxidative damage. Diselenides **13a**–**d** were readily prepared from the diazo compounds **12a**–**d** and were used for the construction of novel *N*-methylated ebselenamines **27a**–**d**. Reduction of **12a**–**d** with PhTeNa prepared in situ produced diselenides **13a**–**d**. Furthermore, the reactions of diselenides **13a**–**d** with four equivalents of MeI in DMSO-*d*6 at room temperature afforded the *N*-methylated ebselenamines **27a**–**d** in moderate yields (Scheme 9). The antioxidant activity of the compounds was evaluated by HPLC-based lipid peroxidation assay, GPx-like activity, and oxidant assays. The *N*-methylated ebselenamine inhibited the peroxidation of linolenic acid, decreased oxidant levels in astroglial cell lines treated with H_2_O_2,_ and mimicked the functions of GPx.

**Scheme 10:**
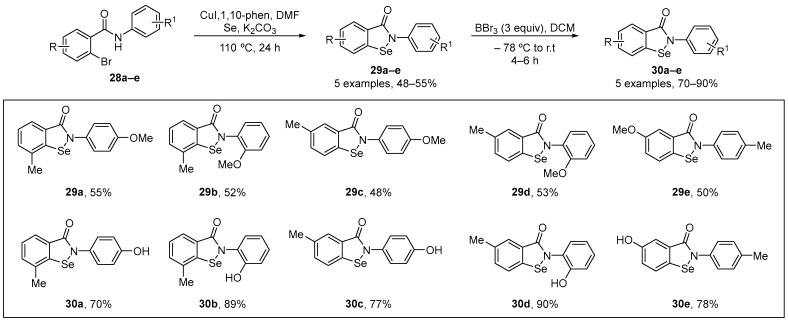
Synthesis of methoxy-substituted isoselenazolones via C-*Se* and *Se*-*N* bond formation.

Singh and co-workers [6] have published a copper-catalyzed direct selenation of substituted 2-bromo-*N*-phenylbenzamide substrates using elemental selenium powder as the Se source to prepare methyl-protected *N*-phenyl substituted isoselenazolones **29** via simultaneous C-*Se* and *Se-N* bond formation. In this process, the substituted phenolic isoselenzolones **30** were generated via a deprotection reaction. After synthesis of the benzamides **28a**–**e**, a copper-catalyzed reaction using CuI and elemental *Se* was performed using K_2_CO_3_ as base and 1,10-phen as a ligand, with this generating the desired isoselenazolones **29a**–**e**. O-demethylation of **29a**–**e**, using stoichiometric amount of BBr_3_ in DCM at −78 °C, gave the phenolic substituted isoselenazolones **30a**–**e** in yields of 70−90% (Scheme 10). The antioxidant activity of the **30a**–**e** was assessed using the FRAP assay. The compounds at concentrations of 10–150 μM showed activity in the FRAP assay, with the activity following the order **30b** > **30a** > **30d** > **30a** > **30e**. GPx mimetic activity of was evaluated in both thiophenol and coupled-reductase assays. All compounds exhibited GPx-like activity, indicating that these phenolic isoselenazolones have promising protective properties.

**Scheme 11:**
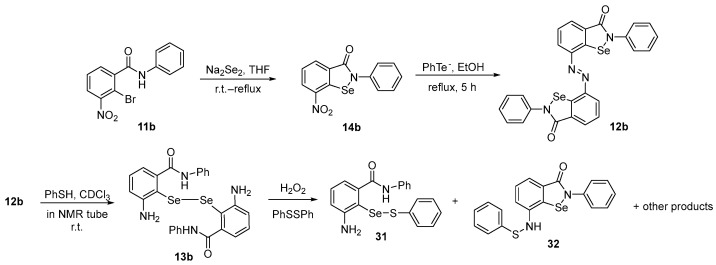
Studies on the oxidation of diselenide **13b**.

To obtain mechanistic insights into intermediates involved in the GPx-like activity of Ebselen derivatives, the azo-bis-ebselen **12b** was prepared by Kumar and Singh [56] using a previously published procedure [47]. Firstly, **12b** was reacted with PhSH in an NMR tube at room temperature in CDCl_3_, being totally reduced to diselenide **13b**. Once formed, **13b** reacts with H_2_O_2_ in the presence of disulfide to form selenenyl sulfide **31**, ebselenamine **14b**, and *N*-thiophenyl-ebselenamine **32** (Scheme 11). It was observed that *N*-thiophenyl ebselenamine **32** and its corresponding selenenyl sulfide **31** were more efficient in catalyzing H_2_O_2_ reduction than Ebselen and mimic the action of GPx.

### 3.3. Se-Functionalized N-Heterocycles

**Scheme 12:**
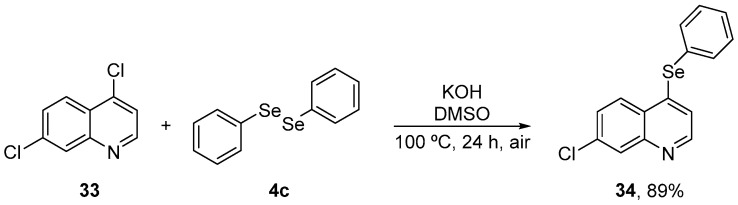
Synthesis of 7-chloro-4-(phenylselanyl)quinoline **34** in basic medium.

7-Chloroquinoline derivatives are biologically active units and display a broad range of pharmacological activities. Due to their importance in a variety of synthetic and natural products, considerable efforts have been directed to the development of new structures based on this scaffold. Vogt et al. [57] have prepared 7-chloroquinoline containing a selenium moiety (**34**) to verify the role of the phenylselanyl group in the antioxidant effect. Compound **34** was synthesized in 89% yield by the reaction of 4,7-dichloroquinoline **33** with diphenyl diselenide **4c**, using KOH as base and DMSO as solvent at 100 °C (Scheme 12). The activity of **34** was evaluated against oxidative stress induced by sodium nitroprusside (SNP) in the brain of mice and compared to the parent compound **33**. Male mice were treated with **34** (50 mg/kg, i.g.) and after 30 min, SNP (0.335 μmol/site, 2 μL, intracerebroventricular (i.c.v.)) was administrated. After 1 h, animals were sacrificed, and the brains were removed for the determination of TBARS, protein carbonyl and NPSH levels, CAT, and GST activities. Compound **34** decreased TBARS and protein carbonyl levels and increased CAT and GST activities, and non-enzymatic NPSH levels. In contrast, **33** did not protect against SNP induced alterations. Therefore, the phenylselenyl group present in the quinoline structure appears to be critical for the protective activities of **34**.

Prigol and co-workers have further investigated the effect of **34** as a multi-target molecule in flies (*Drosophila melanogaster*) [58]. Compound **34** may act on the dopaminergic system through multiple different mechanisms including reducing oxidative damage and improving antioxidant defenses factors, thereby protecting dopaminergic neurons, preventing dopamine depletion, and consequently reversing the behavioral motor deficits induced by rotenone. Adult flies were exposed to a diet containing rotenone (500 μM) and/or **34** (25 μM) for 7 days. The flies were then euthanized, and the heads were removed to determine oxidant and TBARS levels, and SOD, CAT, and NPSH activities. Compound **34** reduced oxidant and TBARS levels and restored the activities of SOD and CAT.

**Scheme 13:**
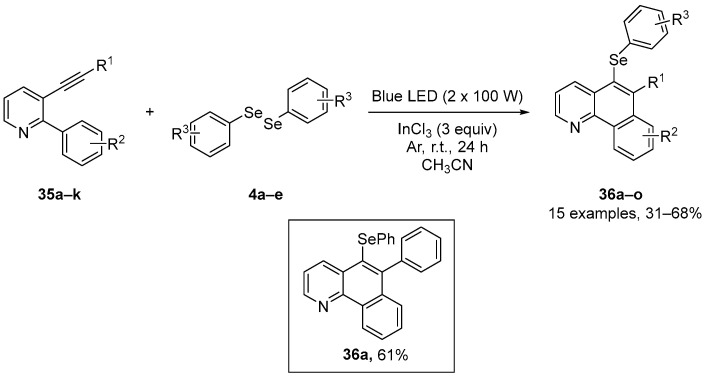
Synthesis of 6-organyl-5-(arylselanyl)benzo[*h*]quinolines **36**.

The biological activities of selenium-substituted quinolones were further examined by Schneider and co-workers [9], who reported a simple and efficient method to access the 6-organyl-5-(arylchalcogenyl)benzo[*h*]quinolines **36**. These were prepared in 40–68% yields through the visible light-promoted 6-endo-dig selenocyclization of 2-aryl-3-(organylethynyl)pyridines **35**, using diaryl diselenides **4** and indium(III) chloride in CH_3_CN under Ar atmosphere for 24 h (Scheme 13). The effects of compound **36a** in vitro was analyzed using the ABTS, DPPH, and TBARS assays. This compound did not show significant activity when compared to the negative control in the ABTS and DPPH assays, but reduced lipid peroxidation induced by sodium nitroprusside (SNP) in the brain at a concentration of 10 µM, and at concentrations over the range 1–200 µM in the liver of mice. These contrasting data illustrate the difficulties in using simple in vitro assays, such as the ABTS and DPPH assays, to predict biological activity, and emphasize the need for in vivo testing.

**Scheme 14:**
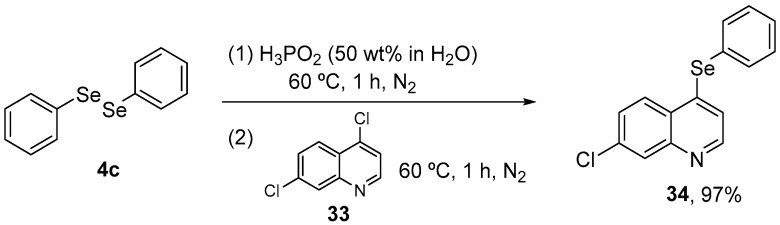
Synthesis of 7-chloro-4-(phenylselanyl)quinoline **34** in acidic medium.

In view of these promising results, Wilhelm and co-workers [59] investigated possible modulation of oxidative stress by **34** in a neuropathic pain animal model. The target molecule was synthesized in 97% yield through the reaction of 4,7-dichloroquinoline **33** with organylselenols, generated in situ by the reaction of diphenyl diselenide **4c** with aqueous H_3_PO_2_ at 60 °C under N_2_ atmosphere (Scheme 14). Adult male mice received streptozotocin (200 mg/kg, i.p.) and after 21 days the animals received **34** (5 mg/kg, i.g.) for 15 days. 24 h after the last treatment, the animals were euthanized and the cerebral cortex, hippocampus, and spinal cord samples were collected for analysis. Compound **34** decreased oxidant levels and SOD, NPSH, GPx, and GR activities in the cerebral cortex and hippocampus, while decreasing oxidation levels and NPSH activity in the spinal cord.

**Scheme 15:**
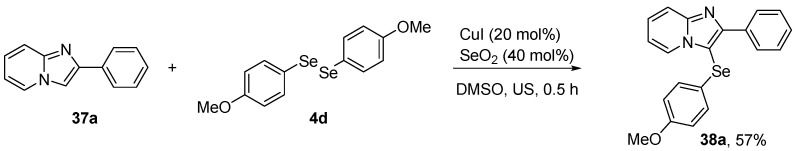
Synthesis of 3-[(4-methoxyphenyl)selanyl]-2-phenylimidazo[1,2-*a*]pyridine (**38a**).

Recent studies have demonstrated the neuroprotective, antidepressant, and antioxidant effects of another organoselenium compound, 3-((4-methoxyphenyl)selanyl)-2-phenylimidazo[1,2-*a*]pyridine (MPI, **38a**). Savegnago et al. [60] have reported the antioxidant activity of **38a** in mouse model of LPS-induced depression. **38a** was synthesized using 2-phenylimidazo[1,2-*a*]pyridine **37a**, (4-OMePhSe)_2_ **4d** and CuI (20 mol%) as copper catalyst in the presence of SeO_2_ (40 mol%) in DMSO under sonication (US) for 30 min (Scheme 15). Male adult mice were treated with compound **38a** (20 and 50 mg/kg, i.g.) 30 min prior the LPS challenge (0.83 mg/kg, i.p.). After 24 h, the animals were sacrificed, followed by brain removal and isolation of prefrontal cortex and hippocampus, for analysis. Pretreatment with **38a** prevented an increase in reactive species and lipid peroxidation induced by LPS in the prefrontal cortex and hippocampus. In related studies, Savegnago and co-workers [61] explored the ability of **38a** to reverse oxidative stress in a mouse model of inflammation, and stress-induced depressive-like behavior. For the inflammatory model, mice received an injection of TNF-α (0.001 fg/site, i.c.v.). After 30 min, they were treated with **38a** (10 mg/kg, i.g.). For the stress model, mice were submitted to physical stress for 240 min. The treatments with **38a** (10 mg/kg, i.g.) were given 10 min after the restraint stress. After 30 min, mice were euthanized for isolation of the prefrontal cortex and total hippocampus for determination of the reactive species, nitrate/nitrite (NOx), and TBARS levels. This treatment with **38a** abolished the oxidative and nitrosative stress in the prefrontal cortex and hippocampus.

**Scheme 16:**
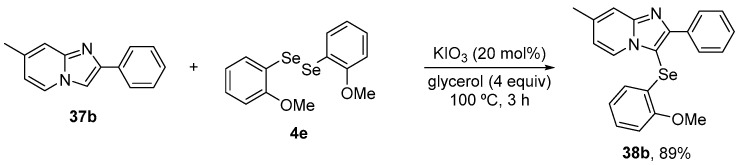
Synthesis of 3-((2-methoxyphenyl)selanyl)-7-methyl-2-phenylimidazo[1,2-a]pyridine (**38b**).

More recently, Ourique and co-workers [62] reported the antioxidant activity and pharmacokinetic characteristics of a related compound, 3-((2-methoxyphenyl)selanyl)-7-methyl-2-phenylimidazo[1,2-a]pyridine (**38b**), in a glioblastoma cell line. **38b** was synthesized by a green protocol, via the direct C(sp^2^)−H bond chalcogenation of imidazo[1,2-*a*]pyridine **37b** with 0.5 equivalent of (4-OMePhSe)_2_ **4e**, using KIO_3_ as a catalyst and a stoichiometric amount of glycerol, giving the target product in 86% yield (Scheme 16). The cells were exposed at low concentration to **38b** (1 μM) for 6 h. After the treatment time, the supernatants were used for TrxR activity, GSH levels, and Nrf2. **38b** modulated the inhibition of TrxR activity and the levels of GSH and Nrf2 proteins.

**Scheme 17:**
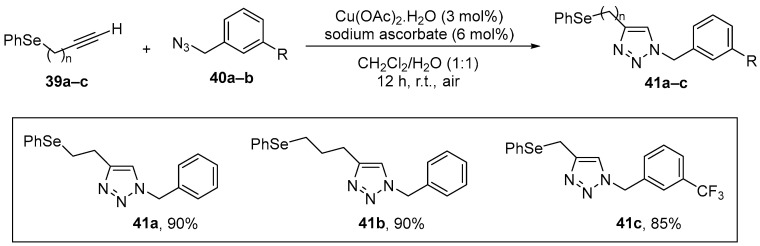
Copper-catalyzed 1,3-dipolar cycloaddition of azidomethyl arylselenides with alkynes.

1,2,3-Triazoles are an important class of nitrogen heterocycles, which display a broad spectrum of chemical applications and biological activities. Ávila and co-workers [10] demonstrated that some variations on this skeleton have the potential to repair oxidative damage by different mitochondrial stress inducers, particularly in a genetic mitochondrial dysfunction model (mev-1 mutation). The selected 4-phenyl-1-(phenylselanylmethyl)-1,2,3-triazole (Se-TZ, **41**) and their derivatives were synthesized in high yield by click chemistry, the copper catalyzed (Cu(OAc)_2_.H_2_O/sodium ascorbate) 1,3-dipolar cycloaddition of azidomethyl arylselenides **39** with alkynes **40** (CuAAC) [63] (Scheme 17). Potential antioxidant effects of **41** were examined in worms (*C. elegans*), which were exposed to the compounds at 10 μM for 30 min at the first larval stage (L1) and then allowed to develop to the L4 stage. Protective effects were examined by the determination of oxidant and NPSH levels and SOD and CAT activities. The compounds reduced the levels of oxidation and CAT activity. In the mev-1 mutants, which have a reduced life span resulting from enhanced mitochondrial oxidant production, treatment with **41** increased the longevity of the worms, possibly through a radical scavenging activity.

**Scheme 18:**
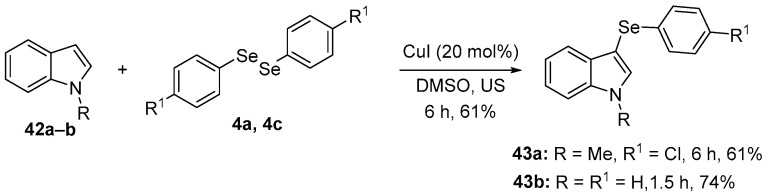
Direct selenation of *N*-methylindole to prepare **43a** and **43b**.

Several studies have highlighted promising protective activities, in different animal models, of *N*-methylindole combined with an organoselenium moiety, and specifically 3-(4-chlorophenylselanyl)-1-methyl-1*H*-indole (**43a**). **43a** was prepared in 61% yield through the direct selenation of *N*-methylindole **42a** with 1,2-bis(4-chlorophenyl)diselenide **4a**, using CuI as catalyst in DMSO under ultrasonic irradiation [64] (Scheme 18). The pharmacological properties of **43a** were investigated by Savegnago and co-workers [65], including in an animal model of neuropathic pain. Male adult mice were submitted to partial sciatic nerve ligation. Four weeks after surgery, the animals were treated with **43a** (10 mg/kg, i.g.) and after 30 min, the mice were euthanized for collection of cortex and hippocampus. Treatment with **43a** decreased oxidant levels and lipid peroxidation (measured using TBARS) in the cortex and hippocampus of the mice. Later studies [66] examined whether **43a** could reverse an oxidative imbalance induced by acute restraint stress in mice. Ten mins after the animals were subjected to 240 min of restraint, they received **43a** (1 or 10 mg/kg, i.g.) with possible protective effects evaluated 30 min later. **43a** reversed oxidative stress in the cortex and hippocampus of stressed mice by reducing oxidant and TBARS levels, and through the modulation of SOD and CAT activities.

Compound **43a** was also examined for its capacity to attenuate neurochemical alterations in a mammary (4T1) tumor model [67]. Female BALB/c mice were subcutaneously inoculated with 4T1 cancer cells (1 × 10^5^ cells/mice). From days 14 to 20, mice received daily gavage with **43a**. Oxidative stress in the prefrontal cortices of tumor-bearing mice was examined by the expression of the NO^•^-producing enzyme iNOS and the transcription factor Nrf2. **43a** decreased the expression of iNOS, increased the expression of Nrf2, and decreased the levels of oxidants, NO^•^, and lipid peroxidation in the prefrontal cortex.

The antioxidant effect of **43a**, and its ability to ameliorate long-term behavioral and biochemical alterations in male mice with sepsis have also been investigated [68]. On day 0, lipopolysaccharide (LPS) (5 mg/kg, i.p.) was administrated to the animals, with treatment with **43a** from days 24 to 30 at 1 mg/kg i.g. After sacrifice on day 30, the prefrontal cortex and hippocampus were removed for analysis. **43a** decreased the effects of sepsis by reducing the levels of oxidants, NO^•^, and lipid peroxidation. This work was subsequently extended [69] to examine antidepressant and anxiolytic effect in animals that received corticosterone (20 mg/kg, i.g.) by 14 days, and on the 15th day, **43a** (1 mg/kg, i.g.). After 30 min, the mice were anesthetized, and blood was collected by cardiac puncture. Treatment with **43a** decreased oxidant levels and lipid peroxidation in the mouse plasma.

Savegnago et al. [70] have also evaluated **43a** against H_2_O_2_-induced oxidative stress in human dopaminergic neuroblastoma (SH-SY5Y) cells. Cells were pretreated with **43a** (4 µM) for 4 h, followed by treatment with H_2_O_2_ (343 µM) for 24 h. The levels of NO^•^ metabolites, oxidants, and GSH were then determined. A decrease in oxidants induced by **43a** treatment was associated with changes in GSH levels, suggesting that the antidepressant and anxiolytic effect of **43a** are related to its antioxidant activity.

1-Methyl-3-(phenylselanyl)-1*H*-indole (**43b**) is another indole-based organoselenium compound that has demonstrated promising antioxidant activity in several animal models of depression and anxiety. **43b** was synthesized in a 74% yield using the same approach used to prepare **43a** (Scheme 18). Male mice received an i.c.v. injection of streptozotocin (0.2 mg/4 μL per mouse). After 24 h, the mice were treated with **43b** (10 mg/kg, i.g.) once daily for seven days; 30 min after the final administration of **43b**, the animals were euthanized, and the cerebral cortex and hippocampus collected for analysis. Treatment with **43b** decreased oxidant levels, metabolites of NO^•^, and lipid peroxidation [71]. Later studies [72] demonstrated that a single administration of **43b** at 10 mg/kg, i.g. was able to induce similar reductions in oxidative stress.

Savegnago and co-workers [73] have also demonstrated that **43b** reduces oxidative stress in cerebral structures, liver, and kidney of streptozotocin-induced diabetic mice. In this study, mice were rendered diabetic by a single injection of streptozotocin (200 mg/kg, i.p.). After 7 days of streptozotocin administration, **43b** (10 mg/kg, i.g.) was administered once a day for 14 days. 24 h after the last dose of **43b,** the animals were euthanized and the hippocampus, prefrontal cortex, liver, and kidney were excised for analysis. Treatment with **43b** inhibited lipid peroxidation in the prefrontal cortex, hippocampus, liver, and kidney of the streptozotocin-treated mice. Together, these studies suggest that **43b** has antidepressant and anxiolytic effects and can also ameliorate the central and peripheral complications caused by diabetes in mice, potentially via its antioxidant activities.

**Scheme 19:**
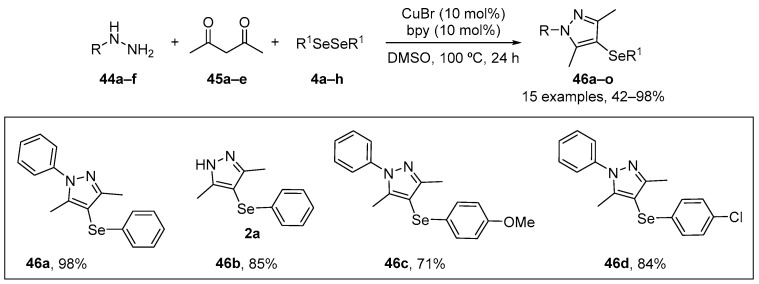
Synthesis of selanyl-substituted pyrazoles **46**.

Oliveira et al. [74] have investigated the antioxidant potential of selanyl-substituted pyrazoles through in vitro and in vivo assays. The compounds were synthesized by a previously described procedure involving a cyclo-condensation and copper-catalyzed direct C-H bond selenation reaction [75]. The one-pot multicomponent reaction of different hydrazines **44**, 1,3-diketones **45** and diorganyl diselenides **4**, using catalytic amounts of copper bromide and bypyridine as ligand afforded a total of fifteen 4-organylselanylpyrazoles **46** in 42–98% yield (Scheme 19). In vitro, the compounds **46a** (50–100 µM), **46c** (100 µM), and **46d** (100 µM) presented FRAP activity, the compound **46a** (10 µM) presented DPPH-scavenging activity, the compounds **46a** (50–500 µM), **46b** (50–500 µM), **46c** (50–500 µM) and **46d** (100–500 µM) inhibited to NO^•^ scavenging, and the compounds **46a** (10–500 µM), **46c** (50–500 µM), and **46d** (50–500 µM) reduced lipid peroxidation and oxidant levels in mouse brain. In vivo, all compounds at concentrations over the range 10–500 µM reduced lipid peroxidation in the liver and brains of mice, consistent with an antioxidant effect of these pyrazoles.

3,5-Dimethyl-1-phenyl-4-(phenylselanyl)-1*H*-pyrazole (**46a**, Scheme 19) was examined by Savegnago et al. in a depression-pain syndrome mouse model induced by streptozotocin [76]. The capability of **46a** to reverse the depression-pain syndrome in mice was examined by evaluating the levels of oxidants, NO^•^, lipid peroxidation, and SOD and CAT activities in the prefrontal cortices and hippocampi of mice. Adult male mice received streptozotocin (0.2 mg/4 μL per mouse, i.c.v.), and after 24 h were treated with **46a** (1 or 10 mg/kg, i.g.). After 30 min, the animals were euthanized and the prefrontal cortex and hippocampus were removed for examination; **46a** treatment decrease oxidant levels, NO^•^ metabolites, and lipid peroxidation in both brain structures. Later studies [77] investigated the anxiolytic-like, antiallodynic, and anti-hyperalgesia effects of **46a** in mice subjected to acute restraint stress (ARS). Adult male mice were restrained for 2 h followed by treatment with **46a** (1 or 10 mg/kg, i.g.). After 30 min, the animals were euthanized, and the prefrontal cortex and hippocampus were removed for analysis. **46a** reversed ARS-induced increased oxidant levels and lipid peroxidation and modulated CAT and SOD activities. Taken together, these studies indicated that the antidepressant-like, antiallodynic, and anti-hyperalgesic effects of **46a** may be related to modulation of oxidative and NO^•^ pathways.

**Scheme 20:**
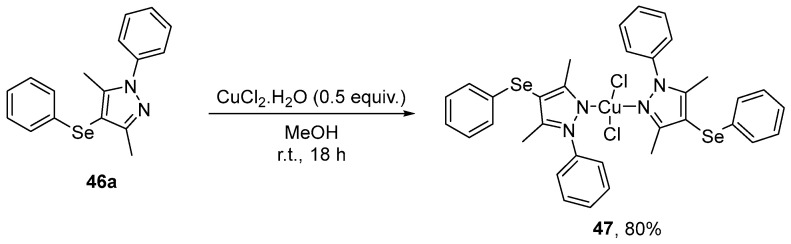
Synthesis of Cu(II) complex **47** derived from **46a**.

Pinheiro et al. [78] have reported the synthesis, characterization, antioxidant potential, and cytotoxicity of a new Cu(II) complex **47**, derived from **46a**. The synthesis involved the slow addition of a solution of **46a** (0.92 mmol) in MeOH to a solution of CuCl_2_⋅2H_2_O (0.46 mmol) in MeOH for 18 h at room temperature, obtaining the desired product in 80% yield (Scheme 20). The antioxidant activity of **47** was evaluated, with concentrations of 10–50 µM shown to inhibit sodium azide-induced formation of reactive species in the hippocampus and cortex of mice. **47** was also effective in scavenging DPPH at different concentrations (1–50 µM), and ABTS^•+^ at 10 µM.

**Scheme 21:**
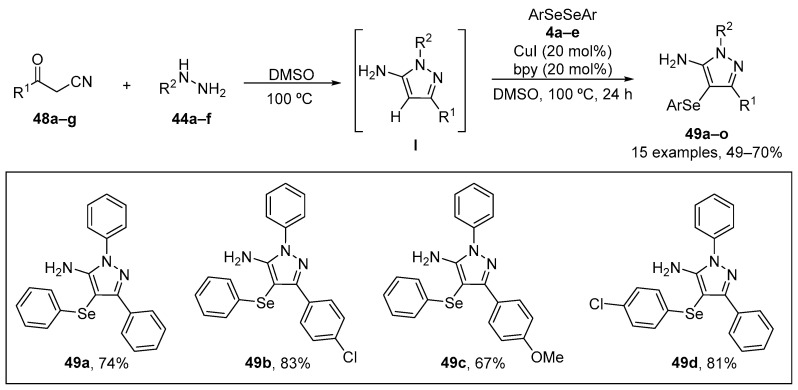
Synthesis of 5-amino-4-(arylselanyl)-1*H*-pyrazoles **49**.

Jacob et al. [79] have reported a sequential one-pot synthesis of 5-amino-4-(arylselanyl)-1*H*-pyrazoles **49** catalyzed by CuI/bpy under mild conditions. The reaction of easily available benzoylacetonitriles **48**, substituted hydrazines **44**, and diaryl diselenides **4** in DMSO at 100 °C for 24 h, afforded a range of 5-amino-4-(arylselanyl)-1*H*-pyrazoles **49** in 49–90% yield (Scheme 21). The antioxidant effect of the 5-amino-4-(arylselanyl)-1*H*-pyrazoles **49** was evaluated in in vitro studies. Compounds **49a**, **49b**, **49c**, and **49d** (50–500 µM) presented reducing power in FRAP assay and the ability to inhibit linoleic acid peroxidation. Compound **49b** was more effective in inhibiting linoleic acid peroxidation than the other compounds, with an IC_50_ value of ~80 µM. Compound **49a** (10–500 µM), **49b** (1–500 µM), **49c** (5–500 µM), and **49d** (10–500 µM) demonstrated ABTS^•+^ radical scavenging activity, with **49a** being the most effective (IC_50_ ~32.5 µM). These 5-amino-4-(arylselanyl)-1*H*-pyrazoles may therefore be worthy of further study. Whether these relative high drug concentrations can be achieved in vivo remains to be established.

**Scheme 22:**
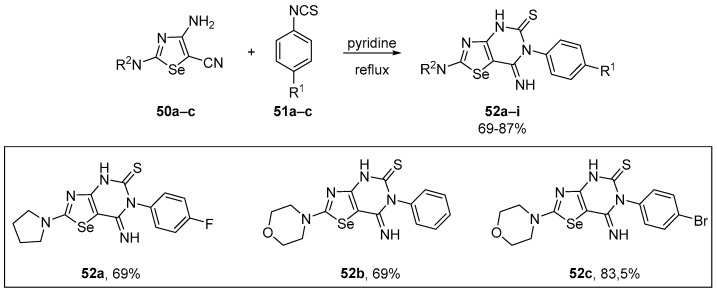
Synthesis of 7-imino[1,3]selenazolo[4,5-d]pyrimidine-5(4*H*)-thiones **52**.

A straightforward strategy for the efficient synthesis of multi-functionalized 7-imino[1,3]selenazolo[4,5-d]pyrimidine-5(4*H*)-thiones **52** was described by Yarmohammadi and co-workers [80]. The target compounds were obtained by the incorporation of the *N*-phenylmethanethioamide fragment through the heteroannulation of several 2,4,5-trisubstituted 1,3-selenazoles **50**. The reaction was conducted using readily accessible phenyl isothiocyanates in pyridine under reflux, and the novel 7-imino[1,3]-selenazolo[4,5-*d*]pyrimidine-5(4*H*)-thione derivatives **52a**–**i** were obtained in 69–87% yield (Scheme 22). The inhibitory activity of the selenium-containing heterocycles was assessed via the DPPH assay, with **52a**, **52b**, and **52c** showing significant activity, with IC_50_ values in the range of 10–63 μM. 4-Fluorophenyl-substituted compounds bearing 2-morpholine (IC_50_ 14 μM), and 2-piperidine (IC_50_ 19 μM) residuals were ranked in the second and third place of antioxidant efficacy, respectively.

### 3.4. Se-Heterocycles

**Scheme 23:**
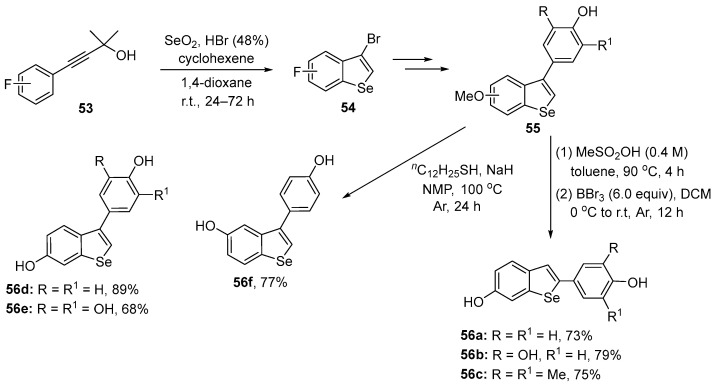
Synthesis of resveratrol-derivative benzoselenophenes **56**.

Arsenyan, Chovanec, and co-workers have reported data for a series of poly-hydroxy 2-aryl- and 3-arylbenzo[*b*]selenophenes **56** and tested their activity in yeast [81]. The compounds were prepared following a procedure described by Arsenyan [82], involving the electrophilic cyclization of arylalkynes **53** with HBr in the presence of SeO_2_, affording the 3-bromo-benzoselenophene **55**, which were converted to the respective 3-aryl derivatives through a Suzuki coupling of **54** with the appropriate arylboronic acid. In the sequence, compounds 2-aryl-substituted **56a**–**c** were obtained by an acid-induced 3,2-aryl shift and deprotection, while **56d**–**f** were prepared after deprotection of the hydroxy groups (Scheme 23).

These resveratrol-inspired benzo[*b*]selenophenes were tested for their capacity to modulate oxidant levels. Compound **56e** displayed the most potent antioxidant activity (12.3% of oxidant levels at the high concentration of 5 mM). The 3-aryl derivative, **56d**, was more effective than the 2-aryl species **56a** in reducing intracellular oxidant levels at equimolar concentrations. Compound **56b** exhibited higher antioxidant activity than **56a**, probably due to the presence of the additional 3’-OH group. Compound **56c** was less effective, and compound **56f** increased oxidant levels when compared to the controls; the reasons for this are unclear.

**Scheme 24:**
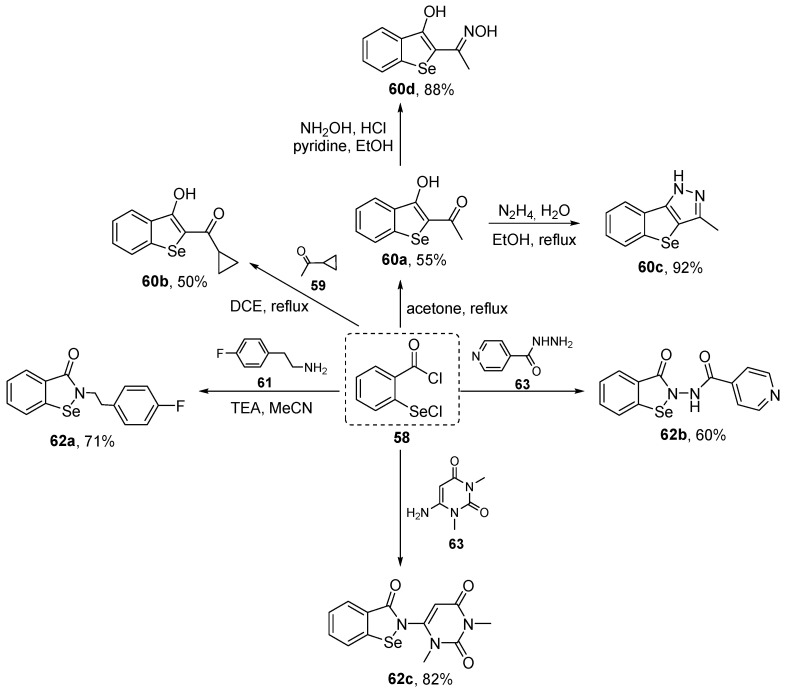
Synthesis of benzo[*b*]selenophenes **60** and Ebselen analogues **62**.

Hamama and co-workers have prepared a series of benzo[*b*]selenophene **60** and Ebselen analogues **62** starting from the versatile intermediate 2-(chloroseleno)benzoyl chloride **58** [83], and evaluated their activity in vitro using ABTS. A total of four benzo[*b*]selenophenes **60** and three Ebselen derivatives **62** were prepared in modest to very good yields by the reaction of **58** with ketones and amines, respectively (Scheme 24). The results obtained from the ABTS showed that **60d** exhibited the greatest activity, with 89% inhibition at a concentration of 2 mM. Compounds **60d**, **60b**, and **62b** at the same concentration showed antioxidant activities similar to that of ascorbic acid, used as a positive control.

**Scheme 25:**
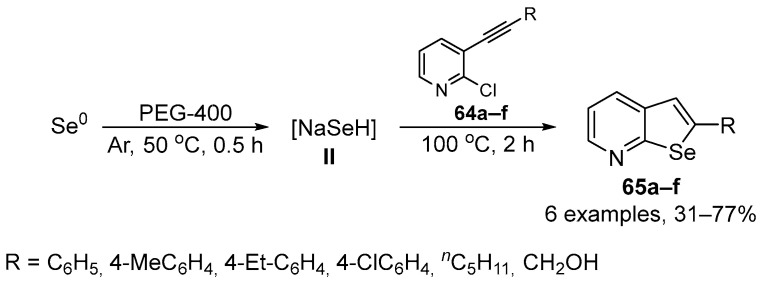
Synthesis of 2-organylselenopheno[2,3-*b*]pyridines **65**.

Perin and co-workers have developed a new general method to prepare 2-organylselenopheno[2,3-*b*]pyridines **65** by the insertion of nucleophilic selenium species **64** into 2-chloro-3-(organylethynyl)pyridines **64** [84]. The selenide anion NaHSe was generated in situ from elemental Se and NaBH_4_/PEG-400 as reducing system, and the nucleophilic attack was followed by an intramolecular cyclization to give the expected selenophene **65**. A total of six selenophene-fused to pyridine ring were prepared in 31–77% yield after 2.5 h of reaction at 50–100 °C (Scheme 25). The antioxidant activity of compounds **65** was evaluated in different in vitro assays of thiols, DPPH and ABTS, and SOD-like activity. Selenophene **65** could reduce hepatic production of oxidants induced by sodium azide in mice at the high concentration of 200 μM. However, **65** did not exhibit significant scavenging activity against DPPH and ABTS^•+^ radicals at any of the tested concentrations, nor demonstrated any SOD-like activity. These results suggest that **60** may have in vivo effects that cannot be modeled by in vitro assays and may not be due to traditional radical scavenging effects.

**Scheme 26:**
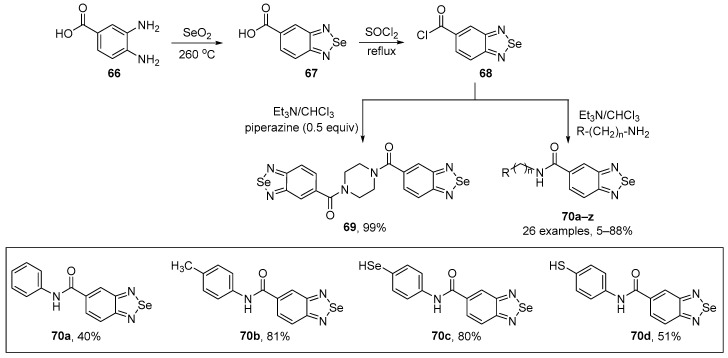
Synthesis of benzo[*c*][1,2,5]selenadiazole derivatives **69** and **70**.

Sanmmartín and co-workers have developed a general method to prepare the benzo[*c*][1,2,5]selenadiazole-5-amide derivatives **69** and **70**, starting from benzo[*c*][1,2,5]selenadiazole-5-carboxylic acid **67** (BSCA) [85]. The key intermediate **67** was prepared from 3,4-diaminobenzoic acid **66** and selenium dioxide under heating, as previously described by the authors [86]. A total of twenty-seven compounds were prepared from the reaction of acyl chloride **68** and different amines in 5–99% yield (Scheme 26). The new selenadiazoles **70a**–**d** were tested for their radical scavenging activity using DPPH. Compound **70d** was the most effective with values exceeding 40% at a concentration of 750 µM. The scavenging capacity was concentration-dependent (0.082 to 828 µM) but not time-dependent over the time frame 30–180 min. Compounds **70a**–**c** also demonstrated potent DPPH radical scavenging activity, with compound **70c** displaying the highest activity, followed by **70b** and **70a**.

**Scheme 27:**
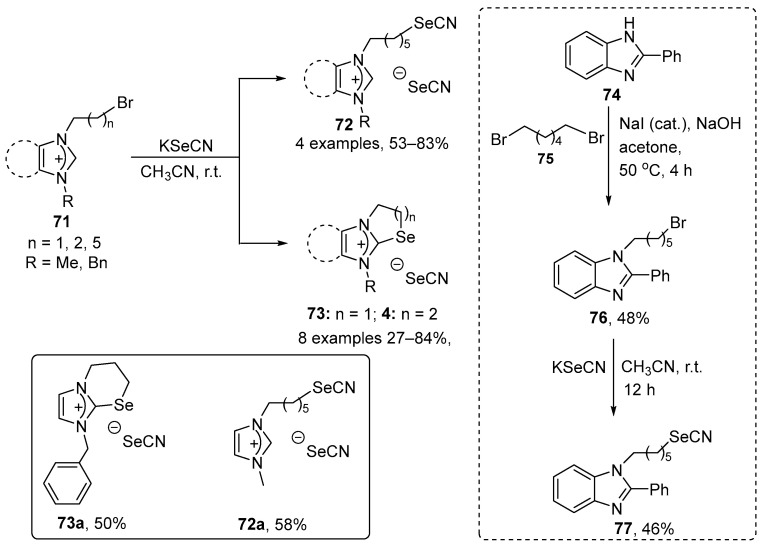
Synthesis of imidazole-fused selenazolium and selenazinium selenocyanates.

Bhabak and co-workers have described the synthesis of new benzimidazole- and imidazole-fused selenazolium and selenazinium selenocyanates (**72**, **73**, and **77**) [87]. Selenazolium and selenazinium selenocyanates **73** were prepared in 27–84% yield through the cyclization of *N*,*N*′-disubstituted benzimidazolium and imidazolium bromides **71** containing *N*-(CH_2_)_2_-Br and *N*-(CH_2_)_3_-Br groups in the presence of KSeCN. In contrast, *N*,*N*′-disubstituted benzimidazolium and imidazolium bromides **71** substituted with *N*-(CH_2_)_6_-Br afforded the open-chain selenocyanates **72** in 53–83% yield. When bromide **76**, derived from 2-phenyl-1*H*-benzo[d]imidazole **76**, was used as stating heterocycle, selenocyanate **77** was obtained in 46% yield after reaction with KSeCN (Scheme 27). The activity of these compounds was evaluated using a GSH–GSSG coupled assay (GPx-like) and a related PhSH–PhSSPh assay. The two compounds were also tested for their capacity to modulate the intracellular level of reactive species in the macrophage-like cell line, RAW 264.7. Most of the compounds displayed significant activity in GPx-like assays. In the GSH–GSSH coupled assay, the reduction of H_2_O_2_, cumene hydroperoxide (Cum-OOH), and tert-butyl hydroperoxide (TBHP) were examined. All the compounds reduced H_2_O_2_ with higher efficacy than Cum-OOH and TBHP, especially compound **73d**. In the PhSH–PhSSPh assay, all the ionic organoselenium compounds exhibited significantly higher activity than the control, with compound **73a** being the best in class. The cyclic selenazolium and selenazinium compounds displayed superior activities to their acyclic analogues. The observed activities of the ionic selenocyanates were determined to be the result of the combined effect of both selenium centers, with a higher contribution from the selenocyanate counter anion. Compounds **73d**, which exhibited the best activity in the GSH–GSSH assay, and **72e,** were also examined for their capacity to remove endogenous H_2_O_2_ in the RAW 264.7 cells. The data obtained indicate that the antioxidant activity of compound **73d** is dose-dependent and higher than that of **72e**.

**Scheme 28:**
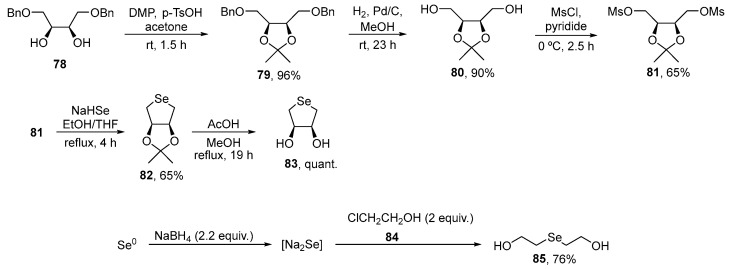
Synthesis of di-hydroxy selenides **83** and **85**.

To examine the role of structural changes in the antioxidant activity of organoselenium compounds, Singh, Priyadarsini, and co-workers prepared cyclic *DL*-*trans*-3,4-dihydroxy-1-selenide **83** and bis(ethan-2-ol)selenide **85** [88]. Both compounds were prepared via previously described procedures. Synthesis of selenolane **83** was achieved starting from dibenzyl ether **78**, derived from *cis*-2-butene-1,4-diol, that was converted to the ketal **81**. After reaction with NaHSe, generated in situ, and deprotection, the cyclic selenide **83** was obtained in 65% yield [89]. The open-chain diol **85**, in turn, was prepared in 76% yield by the reaction of chloroethanol **84** with Na_2_Se, which was obtained in situ from elemental selenium and NaBH_4_ [90] (Scheme 28). The compounds were assayed for their ability to scavenge peroxynitrite (ONOO^−^/ONOOH), hydroxyl (HO^●^), nitrogen dioxide (NO_2_^●^), and carbonate (CO_3_^●−^) radicals.

Compound **83** showed a higher rate constant for reaction with peroxynitrite as compared to the open-chained compound **85**. With CO_3_^●−^, the kinetic studies demonstrated a rate constant of 1.2 ± 0.2 × 10^9^ M^−1^s^−1^ for compound **83** and 6.5 ± 0.3 × 10^8^ M^−1^s^−1^ for **85**. No reaction was observed for **83** and **85** with NO_2_^●^, while the rate constants for reaction with HO^●^ were comparable. The results indicated that cyclic **83** exhibits higher oxidant reactivity than the linear isomer **85**, particularly with peroxynitrite, with a rate constant that was twice as high. These data are consistent with other kinetic data for a range of sulfur and selenium compounds [48] and for enhanced reactivity of 5-membered cyclic species (when compared to 6-membered and acyclic / linear species) [91,92]. The enhanced rates of reaction for the 5-membered ring structures may arise from stabilization of the reaction intermediate formed at the electron-deficient sulfur or selenium center by suitably placed lone pairs of electrons.

These cyclic selenides are related to a family of seleno-substituted sugars that have been previously reported and subsequently tested for biological activity [93]. A range of 5-, 6-, and 7- membered sugar rings have been synthesized with the 5-membered rings containing a Se atom, being kinetically the most favorable synthetic product. All of these species show high reactivity with a wide range of oxidants [49,51,94,95] including H_2_O_2_ alkyl peroxides, hypochlorous acid (HOCl), peroxynitrite, and ^1^O_2_. These reactions generate the corresponding selenoxide in stoichiometric reactions, with no evidence for ring opening or other products. The selenoxides have been shown to be readily re-reduced by both enzymes (e.g., glutathione reductase at the expense of NADPH), and reductants such as GSH, resulting in regeneration of the selenosugar [96]. As a consequence of this (rapid and efficient) recycling, only low concentrations of these species may be required for efficient oxidant removal in vivo. Testing of these compounds in a range of biological assays including cells, isolated aortic rings, and animal models of inflammation have yielded positive data, and these species are currently under development as skin healing agents [93,97,98,99,100] (e.g., against atopic dermatitis) amongst other pathologies.

**Scheme 29:**
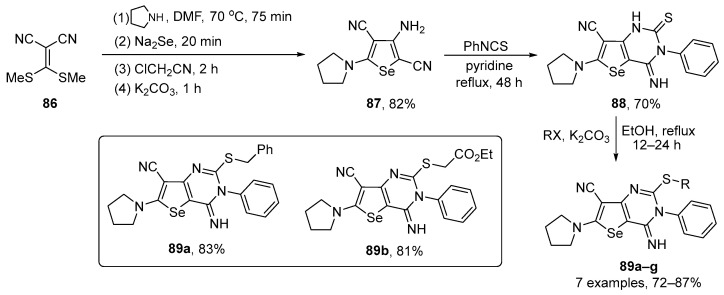
Synthesis of pyrimidine-fused selenophenes **89**.

Shiri and co-workers have prepared several pyrimidine-fused dihydroselenophenes **89** and tested their activities in the DPPH assay [101]. The key intermediate in the synthesis is the pyrrolidine-substituted selenophene **87**, which was prepared in 82% yield from 2-(bis(ethylthio)methylene)malononitrile **86** after four sequential reactions. Once prepared, **87** was reacted with phenyl isothiocyanate in the presence of pyridine to give the pyrimidine-fused selenophene **88** in 70% yield. After reaction with several alkyl halides, seven *S*-functionalized products **89** were obtained in 72–87% yield (Scheme 29). Among the tested compounds, the highest antioxidant activity was observed for compound **87** (IC_50_ ~12 µM), which was much more effective than ascorbic acid, the positive control (IC_50_ ~22 µM). Activity was also detected for the *S*-substituted selenpheno[3,2-*d*]pyrimidines **89a**–**g**, with IC_50_ values in the range 37–54 µM. The order of radical stability is **87** > **89a**–**g** > **88** and this order is probably due to the presence of –NH_2_, –SCH_2_–, and –NHC=S groups acting as hydrogen atom donors, respectively. In the case of **89**, the antioxidant activity is more significant in selenopheno[3,2-*d*] pyrimidines **89a**–**b** (IC_50_ ~37 and ~39 µM), probably due to the presence of adjacent radical stabilizing groups, including Ph and CO_2_Et.

### 3.5. Selenides

**Scheme 30:**
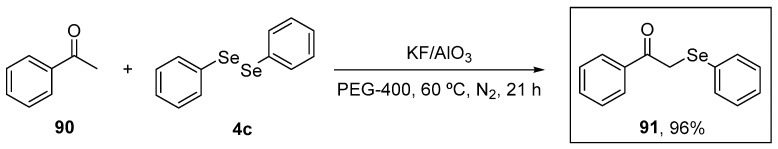
Synthetic route to the synthesis of α-(phenylselanyl)acetophenone **91**.

α-(Phenylselanyl)acetophenone (**91**) was evaluated by Sousa et al. [102] for its capacity to modulate oxidative stress induced by acute stress restriction (ARS) in mice. The target molecule was synthesized in 96% yield, as described previously [103], starting from acetophenone **90** and diphenyl diselenide **4c** in the presence of the catalytic system KF/Al_2_O_3_ and PEG-400 as solvent under N_2_ atmosphere for 21 h (Scheme 30). Animals were treated with compound **91** at a dose of 10 mg/kg for 10 min after the ARS. Then, after 4.5 h, they were evaluated for the levels of lipid peroxidation in the cortex and hippocampus using the TBARS assay. Treatment with **91** decreased MDA levels, as well reactive species production, and the nitrite and nitrate (NOx) levels induced by ARS.

**Scheme 31:**
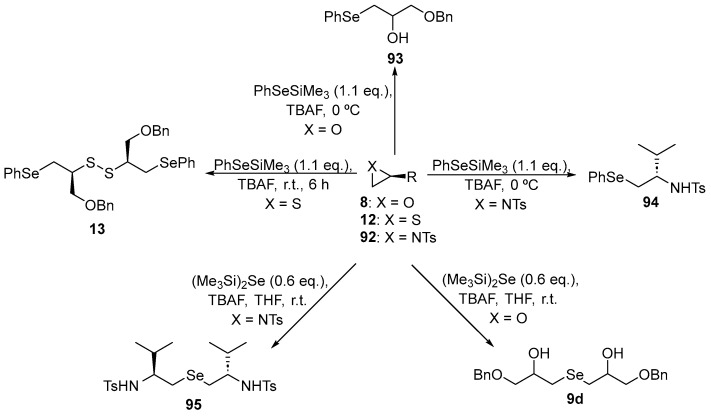
Synthesis of β-functionalized symmetric and non-symmetric selenides.

In order to evaluate the thiol peroxidase-like properties of β-functionalized symmetric and non-symmetric organochalcogenides, Capperucci and co-workers [104] prepared a series of new Se-containing compounds. The β-functionalized selenium compounds were synthesized through the ring-opening of oxiranes, aziridines or thiiranes using trimethylsilyl selenides as nucleophiles (Scheme 31). Catalytic activity was examined in the dithiothreitol (DTT) oxidation model. All the compounds exhibited an equal thiol-peroxidase-like activity of 10.0 mol%. Compound **93** gave the shortest time to reduce the initial thiol concentration by 50% (T_50_ value) after the addition of H_2_O_2_, with a value of ~328 s. The β-hydroxy substituted derivatives **9d** and **93** exhibited superior catalytic properties compared to the β-amino substituted analogs **94** and **95**. Therefore, the substituent at the C-2 position appears to play a crucial role in determining the catalytic properties.

**Scheme 32:**
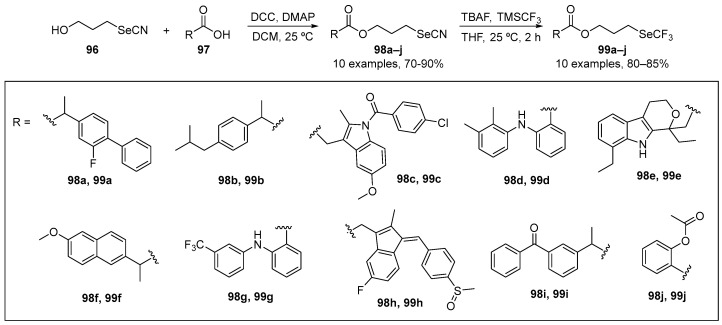
Synthesis of SeCN and SeCF_3_ derivatives.

Zhang and co-workers [105] have reported the synthesis of compounds **98a**–**j** and **99a**–**j** based on the hybridization of nonsteroidal anti-inflammatory drugs (NSAIDs) skeleton and an organoselenium motif (-SeCN and -SeCF_3_). The selenocyanate derivatives **99a**–**j** were obtained by reacting 3-selenocyanatopropan-1-ol with commercially available NSAIDs in the presence of *N*,*N*′-dicyclohexylcarbodiimide and 4-dimethylaminopyridine as condensation agents. The trifluoromethyl selenide derivatives were obtained by reacting the corresponding selenocyanate derivatives with trimethyl(trifluoromethyl)silane (TMSCF_3_) in the presence of TBAF as catalyst to afford **99a**–**j** in yields up to 80% (Scheme 32). The DPPH and GPx-mimetic assays were used to examine the activity of these species. In the DPPH test, compounds **99h** and **99i** (NSAIDs-SeCF_3_ derivatives) were the most active, demonstrating ~45 and ~66% inhibition, respectively. The family of NSAIDs-SeCF_3_ derivatives **99** were more effective than the corresponding NSAIDs-SeCN derivatives **98** (cf. ~17% inhibition **for 98a**, versus ~73% for **99a**). In the GPx-like activity assays, compounds **98h**, **98i**, **99b**, **99e**, **99h**, and **99i** were more active than the other derivatives, with **99h** being the most active mimetic.

**Scheme 33:**
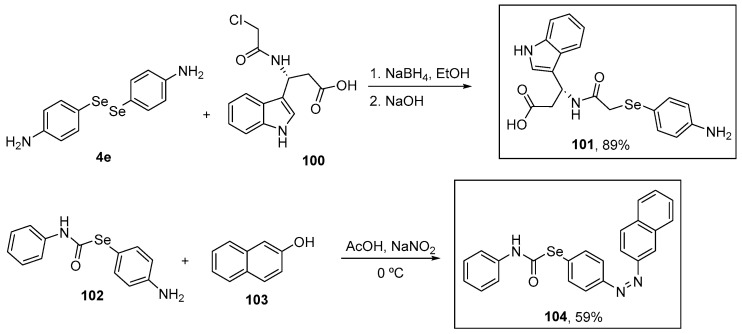
Synthesis of selenide-based azo compounds **101** and **104**.

Shaaban and co-workers [106] synthesized several organoselenium compounds functionalized with amide groups in yields of up to 91%. Compound **101** was prepared in 89% yield from the corresponding 4-amino-substituted diselenide **4e** after reduction under basic conditions (NaBH_4_/NaOH) of the Se–Se bond. The reaction of the intermediate selenolate with alkyl chloride **100** proceeded smoothly at room temperature in only 30 min. A sequential diazotization of 2-((4-aminophenyl)selanyl)-*N*-phenylacetamide **102** and the subsequent coupling with β-naphthol **103** afforded the carbamoselenoate **104** in 59% yield (Scheme 33). Among these OSe compounds, **101** and **104** showed the greatest activity in the DPPH and ABTS assays at (a very high concentration of) 1 mM. In the DPPH assay, compounds **104** and **101** exhibited 93% and 91% inhibition and similar results were found in the ABTS assay with 88% and 85% inhibition, respectively. Compounds **101** and **104** gave IC_50_ values in the DPPH (~24 and ~20 μM, respectively) and ABTS (~32 and ~29 μM, respectively) assay similar to that of ascorbic acid the positive control (DPPH: 19 μM; ABTS: 29 μM).

**Scheme 34:**
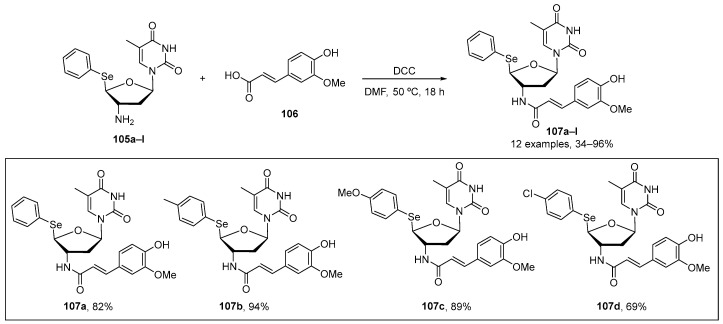
Synthesis of selenium-containing amino-thymide derivatives **107**.

Leal et al. [107] have recently reported the synthesis, antioxidant, and antitumoral activity of new 5′-arylchalcogenyl-3′-*N*-(*E*)-feruloyl-3′,5′-dideoxy-amino-thymidine (AFAT) derivatives. **107a**–**l** were prepared in from good to excellent yields through the reaction of 5′-arylchalcogeno-3′-amino-3′-deoxythymidine (ACAT) **105** with cinnamic acid **106** in the presence of *N*,*N*′-dicyclohexylcarbodiimide (DCC) as coupling agent in dimethylformamide for 18 h at 50 °C (Scheme 34). DPPH and lipid peroxidation (TBARS) assays were used to evaluate antioxidant activity. In the DPPH test, the AFAT derivatives were evaluated at five different concentrations (0.01–1 mM), and all compounds were able to scavenger this radical at the highest concentration. Compounds **107a**–**d** exhibited higher antioxidant activity than their synthetic precursors (**105a**–**d**), particularly compound **107a** and its respective precursor. In the TBARS test, compounds **107a**, **107b**, and **107d** showed significant inhibition, with **107a** having a six-fold higher effect than **105a**. Therefore, fusion of the nucleoside moiety from azidothymidine (AZT) with ferulic acid appears to enhance the antioxidant effects of compounds **105a**–**d** in the TBARS assay.

**Scheme 35:**
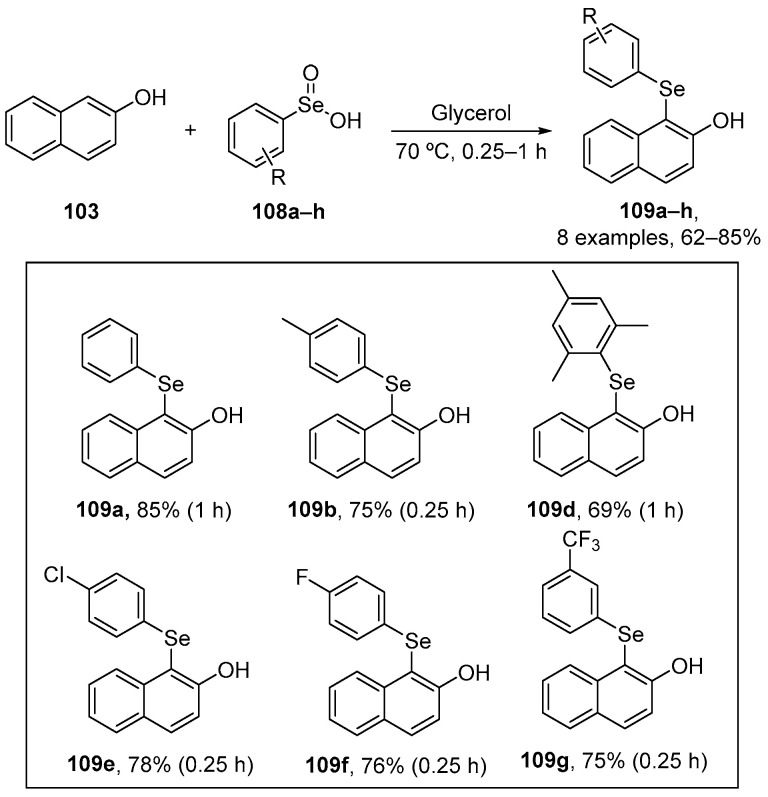
Benzeneseleninic acid in the selenofunctionalization of 2-naphthol derivatives.

In order to develop greener approaches to organoselenium derivatives, Perin and co-workers designed a new protocol to prepare 1-organoselanyl-naphthalen-2-ol **109** through the functionalization of 2-naphthol **103** with arylseleninic acids **108** using glycerol as solvent. The products were obtained in from moderate to good yields (62–85%) [108] (Scheme 35). Antioxidant assays were performed, such as reactive species levels which were measured using DCFH-DA, and DPPH and ABTS to evaluate radical scavenging activity.

Compounds **109a**, **109d**, **109e**, and **109g** were effective in reducing the reactive species production in the liver of mice. Of these, **109d**, **109e**, and **109g** exhibited the best activity, which can be attributed to the mesitylselanyl, *p*-chlorophenylselanyl, and [3-(trifluoromethyl)phenyl]selanyl substituents, respectively. Compound **109d** was effective in the DPPH assay even at 10 μM. Moreover, this compound displayed the highest I_max_ value (~98) and the lowest IC_50_ value (~44 μM), indicating strong antioxidant activity. Compounds **109a**, **109b**, **109e**, and **109f** also scavenged the DPPH radical over the concentration range 100–500 μM. In contrast, compounds **109a**, **109b**, **109d**, **109e**, **109f**, and **109g** demonstrated significant activity against ABTS^•+^ at ≥1 μM. All compounds exhibited high I_max_ values (~95), comparable to the positive control (99.5), and their IC_50_ values were < 5 μM, indicating potent activity. These IC_50_ values were lower than that of ascorbic acid (9.7 μM).

**Scheme 36:**
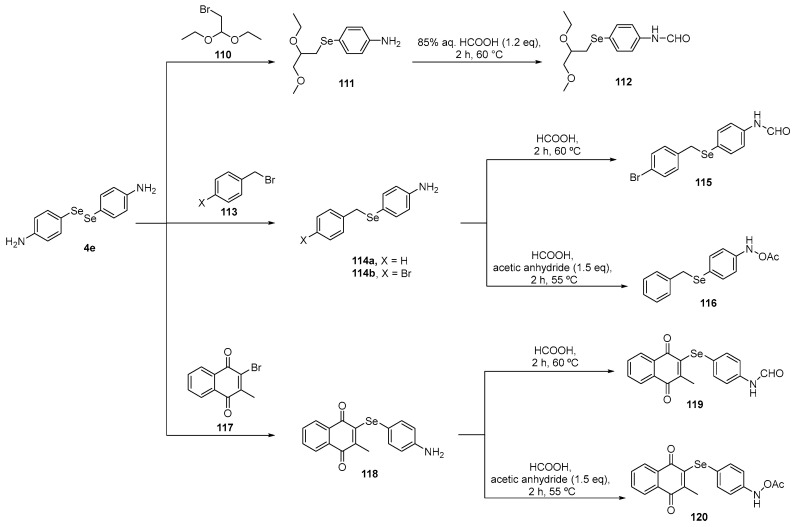
Synthesis of functionalized selenides **114**, **115**, **116**, **119**, and **120**.

**Scheme 37:**
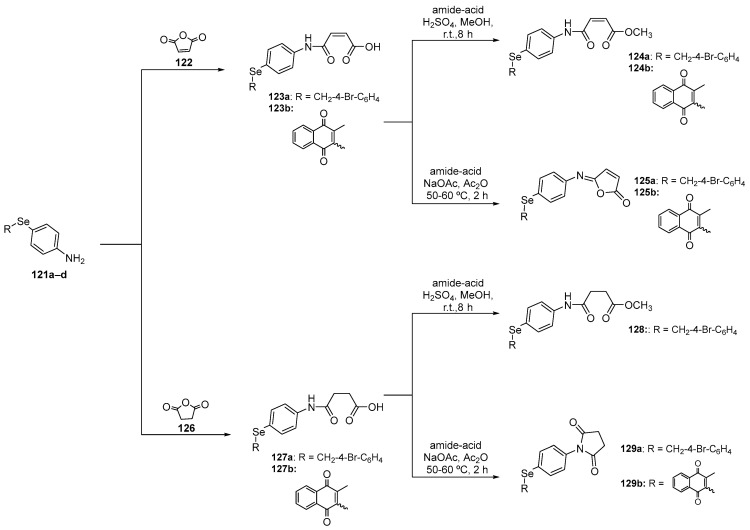
Synthesis of functionalized selenides **124**, **125**, **128**, and **129**.

In a study conducted by Shaaban and colleagues [109], the activity of functionalized benzylselenides and naphthalene 1,4-dione derivatives **114**–**116**, **119**, and **120** was investigated using DPPH, ABTS, reactive species, and GPx-like assays in cultured oligodendrocytes (158N) cells. These compounds were prepared using a starting material 4-amino-substituted diselenide **4c** after a reduction followed by capture of the selenolate intermediate with the respective bromide and reaction with formic acid (Scheme 36). In the same work, the benzylselenides **111** and **114a** were reacted with furan-2,5-dione or dihydrofuran-2,5-dione to prepare, after two steps, the *N*-substituted diselenides **124**, **125**, **128,** and **129** (Scheme 37). In the 158N cell line, most of the compounds reduced reactive species levels, with the rank order in terms of ascending effectiveness at a concentration of 10 µM being **120**, **125a**, **123b**, **127b**, **115**, **114b**, **114a**, **123a**, and **112**.

In contrast to the data obtained for compounds **114**, **115**, **116**, **119**, and **120** (at 10 and 20 μM), selenides **124a**, **128**, and **129a**, as well as the quinone-based organoselenium compounds **124b**, **125b**, and **129b**, caused an increase in intracellular reactive species at all the tested concentrations (10, 20, and 50 μM). Compounds **123b** and **124b**, which are quinoid-based *N*-substituted maleanilic acid and its corresponding methyl esters, exhibited significant GPx-like activities at a concentration of ~40 μM. These compounds were 1.5-fold more active than Ebselen. The other compounds showed moderate GPx-like activity, especially **119d**, **117a**, **128**, and **125b**. Most of the compounds showed good to moderate activity in the DPPH and ABTS assays, except **119d**, **120a**, **120b**, **123b**, **127b**, **124b**, **125b**, and **129b**, which demonstrated pro-oxidant effects.

**Scheme 38:**
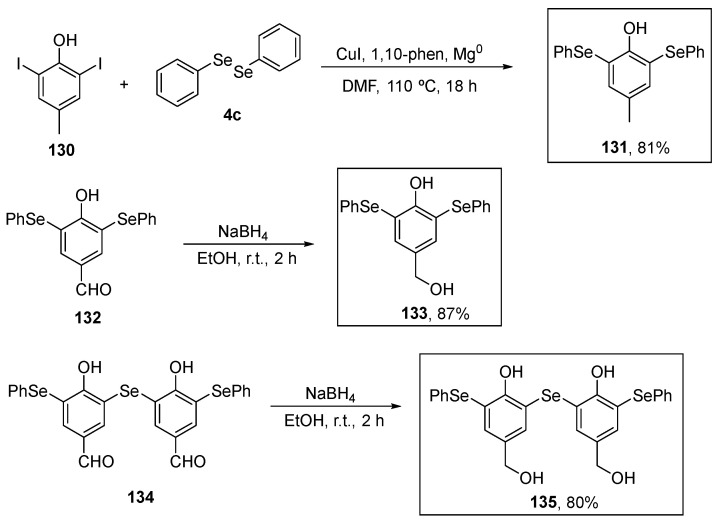
Synthesis of phenol-substituted selenides **131**, **133**, and **135**.

Upadhyay and co-workers (2021) [8] investigated the antioxidant activity of bis- and tris-selenol-bisphenols using the DPPH assay. The target compound **131** was prepared in 81% yield by a new copper(I)-catalyzed reaction of 2,6-diiodophenol in the presence of Mg^0^ in DMF with diphenyl diselenide. Compounds **133** and **135**, in turn, were prepared in 87% and 80% yield by the reduction of the respective aldehydes **132** and **134** (Scheme 38). In the DPPH test, selenophenols **131**, **133**, and **135** exhibited significant antioxidant activity at a concentration of 6.4 μM, surpassing that of the positive control (vitamin E; 6.4 μM). These compounds displayed maximum rate constants of 0.71 ± 0.269 min^−1^, 0.78 ± 0.08 min^−1^, 1.3 ± 0.06 min^−1^, and 1.2 ± 0.13 min^−1^, respectively. In the thiol peroxidase assay, the biselenophenols and tris-selenol-bisphenols **133** and **135** demonstrated reduction rates for H_2_O_2_ of 4.6 ± 0.62 and 13.7 ± 1.17 μM min^−1^, respectively. Interestingly, although **133** and **135** exhibited a lower rate of H_2_O_2_ decomposition, they were able to decompose H_2_O_2_ over significantly longer incubation periods. Lastly, compounds **133** and **135**, along with vitamin E, were assessed for their ^1^O_2_ quenching activity. The tris- selenophenol **135** exhibited a modest reduction in ^1^O_2_ levels, similar to that observed for vitamin E. Therefore, the presence of intramolecular Se^….^O interactions involving the phenolic OH group appears to enhance the oxidant removal activity of these compounds (Scheme 38).

### 3.6. Miscellaneous

**Scheme 39:**
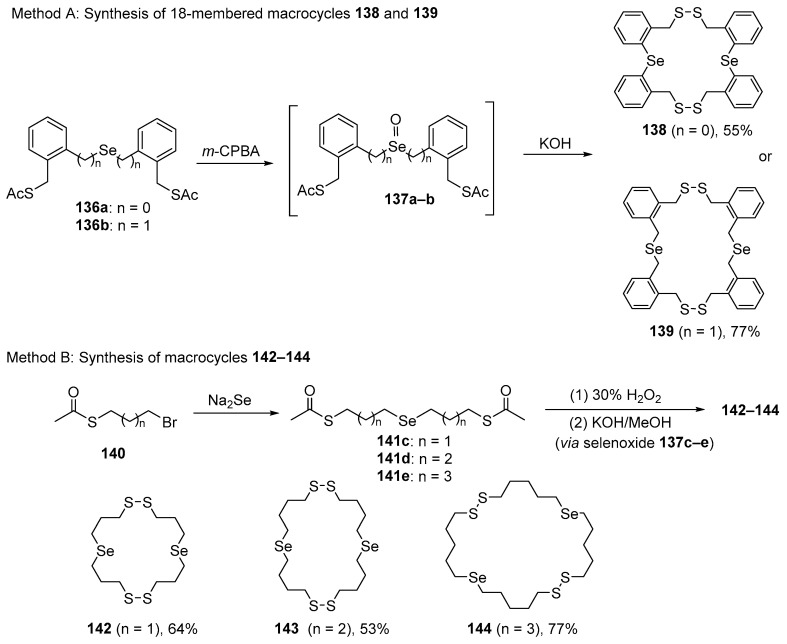
Synthesis of macrocycles **138**, **139**, and **142**–**144**.

A new class of dimeric macrocycles of 18–26 members with potent GPx-like activity in vitro has been reported by Back and co-workers, while they were trying to prepare spirodithiaselenuranes [110]. The unexpected macrocycles **138**–**144** were obtained through the base-promoted oxidative cyclization of selenoxide thioacetates **136a**–**e**, which were generated in situ from the respective selenides **136** (Scheme 39). The novel organochalcogen-embedded macrocycles containing two selenide and two disulfide moieties were able to catalyze H_2_O_2_ reduction at the expense of a thiol. The dimeric macrocycles **139** and **142**–**144** showed the best activity in the GPx-mimetic assay. Kinetic plots obtained for the aliphatic macrocycles **139** and **142**–**144** (10 mol%) revealed that the reactions are remarkably rapid, reaching 50% completion in 2.4 and 4.8 min. Another feature is the considerable induction period for compounds **139** and **142**–**144**, with very rapid consumption of thiol within 7 min.

**Scheme 40:**
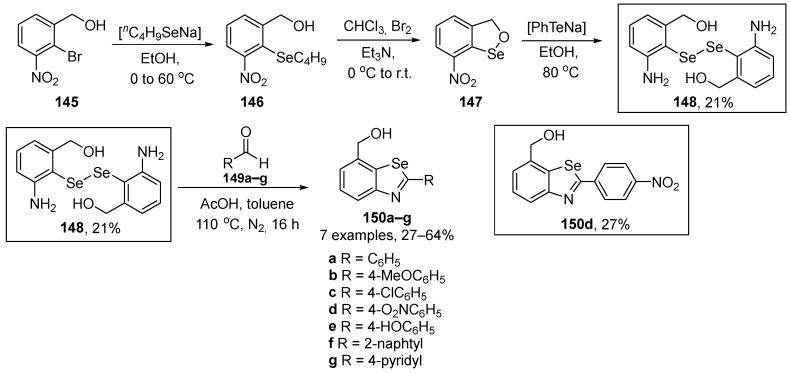
Synthesis of diselenide **148** and benzoselenazole **150**.

Singh and co-workers have described the synthesis, reactivity, and the GPx-like activity of bis(3-amino-1-hydroxybenzyl)-diselenide **148** and its benzoselenazole derivatives **150** [111]. The key intermediate is the diselenide **148**, with two *ortho* groups at the benzene ring (NH_2_ and CH_2_OH). This diselenide was prepared by the reaction of 7-nitro-3*H*-2,1-benzoxaselenole with PhTeNa, generated in situ in 21% yield after three steps. Once obtained, it was easily converted to the respective benzoselenazoles **150** through the one-pot acetic acid-catalyzed reaction with aldehydes (Scheme 40). All the prepared compounds (**148** and **150a**–**g**) presented superior GPx-like activity when compared to diphenyl diselenide and Ebselen, with compounds **148** and **150a** being the most active.

In the GPx-like assay using a thiophenol for the reduction of H_2_O_2_ in the presence of PhSH, the compound **148** (11.5 ± 0.4 μM min^−1^) showed better catalytic activity than Ph_2_Se_2_ and Ebselen, probably due to the presence of the two *ortho* groups to the Se atom. Moreover, the presence of weak secondary Se^…^H interactions and the various intermediates (selenol, selenenic acid, selenylsulfide) formed during the catalytic cycle from compound **148** are likely to give rise to its high GPx-like activity. Compounds **150a**–**g** demonstrated reduction rates higher than those of Ebselen (1.3 ± 0.1 μM min^−1^). There was a trend of increasing reduction rates along the order **150e** < **150g** < **150f** < **150c** < **150a** < **150b** < **150d**. However, the catalytic activity of compounds **150a**–**g** was low, as only one intermediate (selenoxide) was formed during its catalytic cycle.

**Scheme 41:**
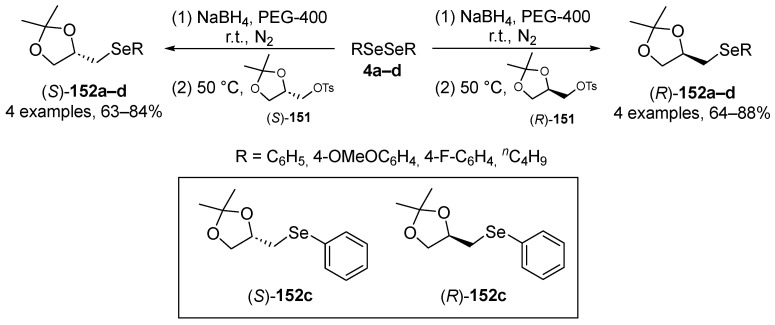
Synthesis of chiral selenides (*R*)-**152** and (*S*)-**152**.

Perin and co-workers have described the synthesis of enantiomerically pure organoselenium compounds (*R*)-**152** and (*S*)-**151**, derived from glycerol, which were tested for their in vitro and in vivo (*C. elegans*) antioxidant activities [112]. The seleno derivatives were prepared through the reaction of chiral solketal tosylates (*R*)-**151** and (*S*)-**151** with selenolate anions, which were generated in situ by reaction of the respective diselenide with NaBH_4_ (Scheme 41). The most active compounds were the selenide (*R*)-**152c** and (*S*)-**152c**, derived from diphenyl diselenide **4c**. The methodology was successfully extended to sulfide and telluride analogues.

The authors have also tested these compounds in multiple in vitro assays, including linoleic acid peroxidation assay, ABTS^•+^, and DPPH scavenging activity, FRAP, chelating potential assay, quantification of reactive species assay, and SOD-like activity. The (*R*)-**152c** and (*S*)-**152c** enantiomeric compounds inhibited the formation of MDA from linoleic acid. Furthermore, in the sodium azide-induced reactive species test, the compounds decreased reactive species in the cortex of mice (5 μM—(*R*)-**152c**; 10 μM—(*S*)-**152c**). However, they did not show activity against DPPH and ABTS^•+^ radicals, the FRAP assay, ion chelation potential, or SOD-like activity. In the in vivo assay, the authors tested (*R*)-**152c** and (*S*)-**152c** in *C. elegans* against added H*_2_*O_2_. *C. elegans* pretreated with sublethal doses of (*R*)-**152c** and (*S*)-**152c** (1 μM and 50 μM) were completely protected against H_2_O_2_-induced mortality. This protection against H_2_O_2_ toxicity may be due, in part, to an increase in CAT enzyme levels observed in animals treated with 1 μM (*R*)-**152c**. Together, these data reinforce the conclusion that in vivo assays are of great value and confirm that data from in vitro assays do not always predict in vivo activity.

**Scheme 42:**
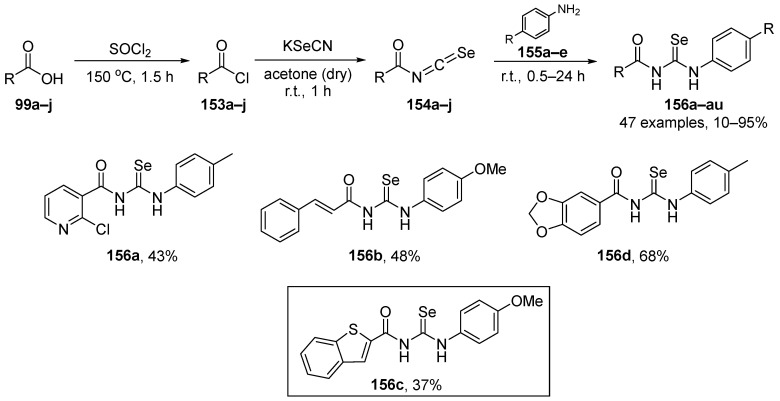
Synthesis of acyl-selenoureas **156**.

Sanmartín and co-workers have prepared new *N*,*N*’-disubstituted acylselenoureas, which were assayed for in vitro DPPH and ABTS^•+^ assay activity, and for protective effects against H_2_O_2_-induced oxidative stress in HT-29 cells [113]. A total of forty-seven selenoureas **156** were prepared and tested in vitro; the four most active ones (**156a-d**) were evaluated in cell experiments. The best in class was the compound **156c**. The *N*,*N*’-disubstituted acylselenoureas **156** were prepared in 10–95% yield from the respective carboxylic acids, after a sequential halogenation-isoselenocyanation-amidation (using aniline) (Scheme 42).

The acylselenourea derivatives show excellent activity in the ABTS^•+^ and DPPH assays (0.77 to 85 µM), with a fast kinetic behavior. In general, phenyl and furyl derivatives are globally the most active with mean DPPH radical inhibition of ~30% at low concentrations. However, ABTS^•+^ radical scavenging activities of acylselenoureas containing pyridyl (**156a**, 8.5 µM), cinnamyl (**156b**, 8.4 µM), benzothienyl (**156c**, 7.7 µM), or benzodioxyl (**156d**, 8.3 µM) showed greater antioxidant capacity at low concentration (~0.0007 µM). Furthermore, the acylselenourea **156c** (benzothienyl) at 77 µM protected HT-29 cells against H_2_O_2_-mediated damage, increasing cell survival by up to 3.6-fold.

**Scheme 43:**
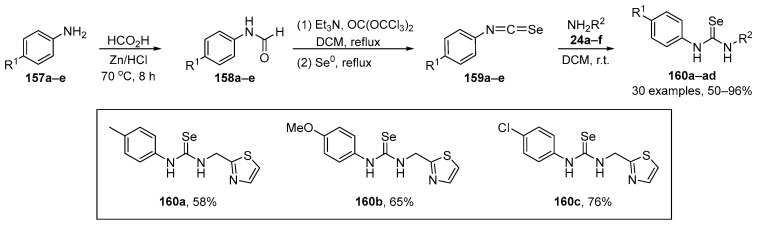
Synthesis of selenoureas **160**.

The same group have synthesized 30 related *N*,*N*′-disubstituted selenoureas **160** containing a *para*-substituted phenyl ring with different electron-withdrawing and electron-donating groups, and different aliphatic and aromatic nuclei [114]. The synthetic strategy to prepare **160** starts with the preparation of formamide **158** through the Zn/HCl-catalyzed reaction between *para*-substituted anilines **157** and formic acid. Once prepared, **158** was converted to the key isoselenocyanate intermediate **159**, by sequential reaction with triphosgene, OC(OCCl_3_)_2_, in Et_3_N, and selenium powder (Scheme 43). All the prepared compounds showed positive effects in the DPPH and ABTS assays, with **160a**, **160b**, and **160c** showing greater activity than the reference substance (ascorbic acid) around 0.00092 µM.

**Scheme 44:**
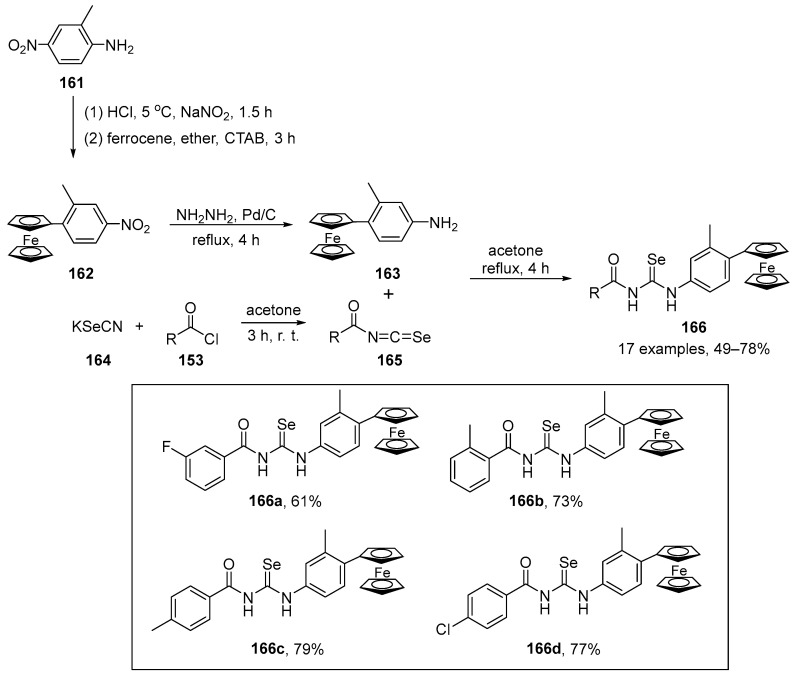
Synthesis of ferrocene-functionalized selenoureas **166**.

Hussain et al. have prepared a new class of selenoureas **166** having ferrocene and substituted benzoyl functionalities with antioxidant activity in the DPPH test [115]. A total of 17 compounds were prepared by a convergent synthesis starting from *p*-nitrotoluidine **161** and KSeCN. In the end of each reaction pathway, 3-methyl-4-ferrocenylphenylaniline **163** reacts with the acyl-isoselenocyanante **165** in acetone to afford the desired selenoureas **166** in 49–78% yield (Scheme 44). Among the prepared compounds, in the DPPH test *ortho*-substituted derivatives (i.e., 2-fluorophenyl derivative) presented higher scavenging activities than *meta*- and *para*-fluorophenyl (i.e., 3-fluorophenyl and 4-fluorophenyl) and substituted derivatives (i.e., methylphenyl derivatives). Compounds **166a**–**d**, which showed >50% scavenging activity, have IC_50_ values of ~152, ~385, ~166, and ~84 μM for **166a**, **166d**, **166b**, and **166c**, respectively, compared to ~55 μM for ascorbic acid.

**Scheme 45:**
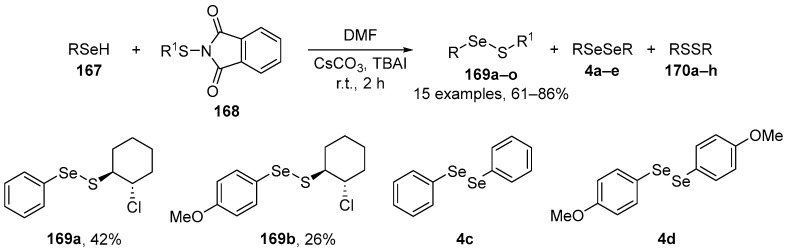
Synthesis of selenylsulfides **169**.

Looking for new GPx-mimic small molecules, Tanini et al. have developed a new approach to prepare selenylsulfides **169** [116]. Fifteen compounds were prepared in 61–86% yield through the reaction of alkyl or arylselenols **167** with *N*-(organylthio)phthalimides **168** in DMF. In some examples, CsCO_3_ and tetrabutylammonium iodide (TBAI) were used as additives and the respective diselenides **4** were obtained as co-products in almost all reactions (Scheme 45). The authors experienced difficulties in isolating the prepared compounds, which quickly disproportionated to diselenide and disulfide. In this context, the selenylsufides (*S-Se* bond) resemble the key intermediates involved in the GPx catalytic cycle, especially compounds **169a** and **169b**. In the GPx-like assay, the presence of a methoxy group in the arylselanyl moiety renders selenylsulfide **169a** more efficient than **169a** (10 mol%). Furthermore, diselenides **4a** and **4c** promote a faster oxidation than selenenylsulfides **169** (ratio of DTT_red_ with 10 mol% of the compounds).

**Scheme 46:**
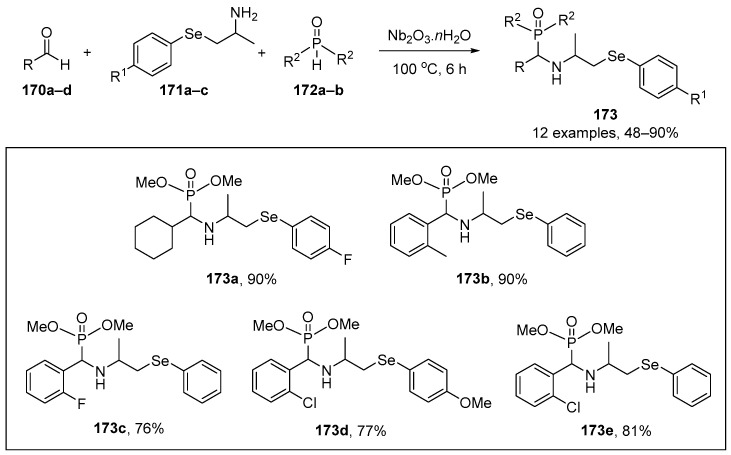
Synthesis of organoselanyl α-amino phosphonates **173**.

Silva and co-workers have described a new approach to organoselanyl α-amino phosphonates **173** through the Kabachnik–Fields multicomponent reaction (MCR) [117]. In this MCR, aldehyde **170**, arylselanyl-2-propylamine **171**, and diorganyl phosphite **172** were reacted in the presence of niobium oxide at 100 °C for 6 h, affording a total of twelve differently substituted organoselanyl α-amino-phosphonates **173** in 48–90% yield (Scheme 46). Six of the prepared compounds were examined for antioxidant assessment in vitro, with compounds **173a**, **173c**, **173d**, and **173e** (500 μM) reported to decrease lipid peroxidation induced by sodium nitroprusside. Compounds **173a** (100 μM) and **173b** (100 μM) showed significant ABTS^•+^ radical-scavenging activity. However, the compounds did not react with DPPH. The activity of these α-amino phosphonates compounds therefore appears to be dependent on their chemical structure.

**Scheme 47:**
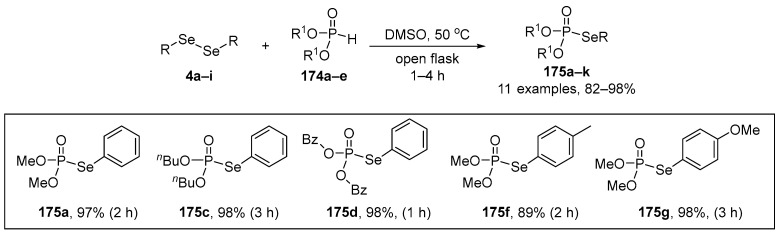
Synthesis of phosphoroselenoates **175**.

The group have described the synthesis of new organophosphate compounds, the phosphoroselenoates **175** [118]. Eleven compounds were prepared in 82–98% yield, by the reaction between diorganyl diselenide 4 and diorganyl H-phosphonate **174** in DMSO at 50 °C for 1–4 h (Scheme 47). Five of the species (**175a**, **175c**, **175d**, **175f**, **175g**) were screened by in vitro (TBARS, reactive species in the brain, FRAP, DPPH, and ABTS^•+^) and ex vivo (lipid peroxidation, SOD and GPx activities in the brain) assays for activity. The compounds showed in vitro antioxidant activity (DPPH scavenging: **175a**, and **175g**) and ABTS^•+^ scavenging: **175a**, **175f**, and **175g**), as well as FRAP activity (**175c**, **175d**, **175f**, and **175g**). The mechanism of action by which phosphoroselenoates present antioxidant activity is related to the reduction of lipid peroxidation (**175c**, **175d**, **175f**, and **175g**) and reactive species (**175a**, **175c**, **175d**, and **175g**). It was demonstrated that, in ex vivo assays, at a dose of 10 mg/kg, compounds **175f** and **175g** reduced lipid peroxidation levels in the brain of mice, while compounds **175a**, **175c**, and **175g** decreased GPx activity, and **175f**–**g** decreased SOD activity.

**Scheme 48:**
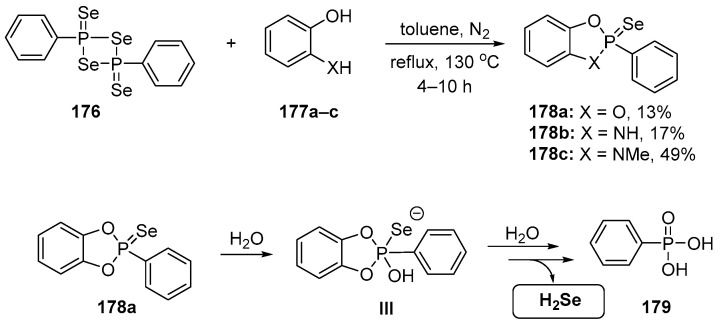
Synthesis of cyclic *P*–*Se* compounds **178** and the releasing of H_2_Se from **178a**.

An alternative strategy to deliver the reactive selenium species was developed by Pluth and co-workers [119]. The authors have prepared three cyclic P–Se compounds **178** and evaluated their ability to release H_2_Se in the presence of water. The P–Se compounds were prepared through the reaction between the Woollins’ Reagent **176** and an *ortho*-substituted phenol **177** (catechol, 2-aminophenol, and *N*-methyl-2-aminophenol). The reaction was conducted under an atmosphere of N_2_ in refluxing toluene for 4–10 h, affording the expected products **178a**, **178b**, and **178c** in 13%, 17%, and 49% yield, respectively (Scheme 48). The authors demonstrated H_2_Se release in the presence of water, which acts as an antioxidant, as demonstrated in liver cells (HeLa) treated with H_2_O_2_. In general, **178b** demonstrated antioxidant activity toward exogenous (concentration of 25 μM) and endogenous (>5 μM) oxidants, which suggests that this compound exerts antioxidant effects on cells.

**Scheme 49:**
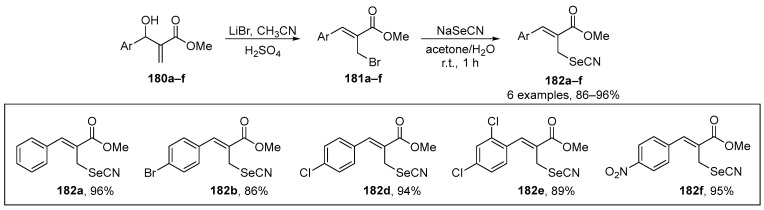
Synthesis of allyl selenocyanates **182**.

Selenocyanates are an interesting class of organoselenium compounds which contain the SeCN unit. Rafique and co-workers have prepared a new class of selenocyanates derived from α,β-unsaturated esters [120]. The most active antioxidant compounds were those having *para*-substituents in the phenyl ring (4-Br, 4-Cl, and 4-NO_2_). The synthesis of six methyl (*Z*)-3-aryl-2-(selenocyanatomethyl)acrylates **182** was achieved in two steps from the Morita–Baylis–Hillman adducts α-methylene-β-hydroxy esters **180**. Firstly, **180** was converted to the respective allyl bromide **181**, followed by the reaction with NaSeCN in a 4:1 mixture of acetone:H_2_O at room temperature for 1 h, to give **182** in 86–96% yield (Scheme 49).

The authors evaluated the antioxidant activity of the compounds using different assays, including TBARS, 4-HNE, and 8-isoprostane (8-ISO) tests for the assessment of lipid peroxidation parameters. They also used the 8-hydroxy-2’-deoxyguanosine (8-OHdG) test to assess DNA damage, and the carbonyl test for the assessment of protein oxidation in cultured mouse neurons exposed to H_2_O_2_ and treated with **182**. CAT and SOD activities were also evaluated. The cells exposed to H_2_O_2_ in culture containing the compounds **182b**, **182d**, and **182f** (10 μM) exhibited the most pronounced pattern of antioxidant activity in TBARS, 4-HNE, 8-ISO, 8-OHdG, and carbonyl levels. These results were similar to those of diphenyl diselenide. Compounds **182a**–**f** showed significant attenuation of CAT activity. Moreover, compounds **182a**–**d** upregulated SOD activity in the neurons exposed to H_2_O_2_.

**Scheme 50:**
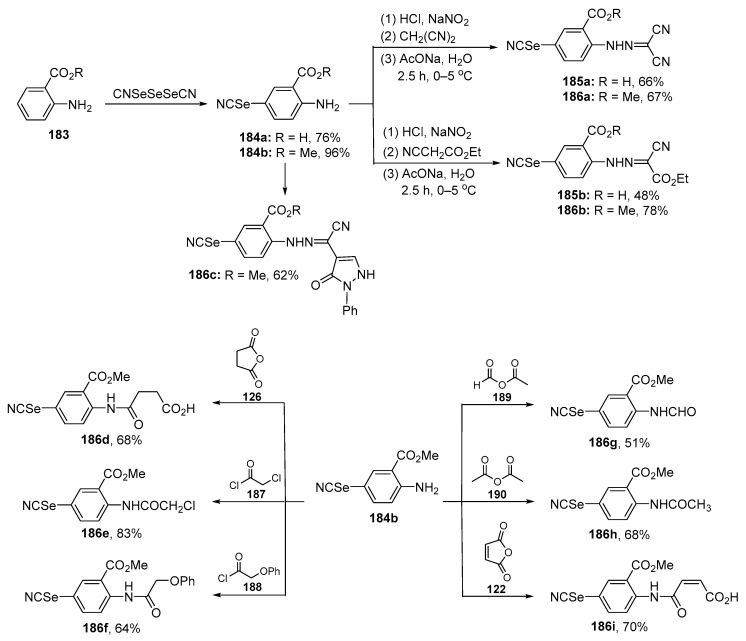
Synthesis of selenocyanates **184**, **185**, and **186** derived of anthranilic acid.

More recently, Shaaban and co-workers described the synthesis and in vitro antioxidant activity (DPPH and ABTS^•+^) of new organoselenocyanates **185** and **186**, derived from anthranilic acid **183** [121]. The key intermediates for the synthesis were 2-amino-5-selenocyanatobenzoic acid **184a** and its methyl ester **184b**. After several reactions at the amino group, the derivatives **185a**–**b** and **186a**–**i** were prepared in 51–83% yield (Scheme 50). The organoselenocyanate compounds (1 mM) demonstrated scavenging activities of both ABTS^•+^ and DPPH radicals similar to ascorbic acid (positive control). Among the tested compounds in the DPPH assay, **184b** exhibited the highest scavenging activity, followed by **186c**, **186b**, **186f**, **185a**, and **186g**. In the ABTS test, the most reactive species was **186c** followed by **186f**, **184b**, **185b**, **186g**, and **185a**.

**Scheme 51:**
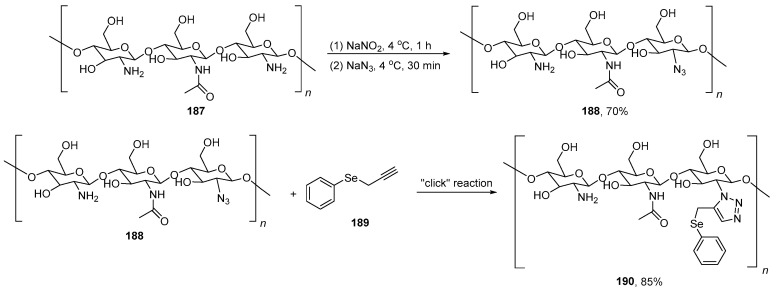
Synthesis of selenium-containing chitosan **190**.

Chitosan (CS) **187**, a natural polysaccharide, was used by Fajardo and co-workers as a starting material to prepare the organoselenium-chitosan derivative **190** [122]. The key intermediate in the reaction was the chitosan-azide **188**, which was the substrate in the “click” reaction with 3-(phenylseleno)prop-1-yne **189**, affording the expected triazole **190** in 85% yield (Scheme 51). Compound **190** demonstrated activity against ABTS^•+^ radicals in a concentration-dependent manner (≥3.0 g L^−1^). The maximum inhibition (I_max_) achieved by compound **190** was 33.5%. Additionally, compound **190** exhibited significant DPPH scavenging activity at concentrations ≥1.0g L^−1^, with an I_max_ of 40.0%.

**Scheme 52:**
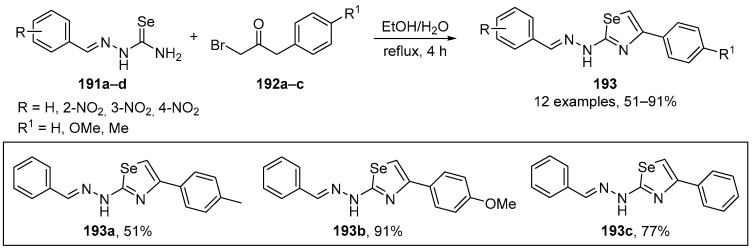
Synthesis of selenazolyl-hydrazones **193**.

Filipovic and co-workers have prepared selenazolyl-hydrazones **193**, which were tested in in vitro assays (DPPH, TRP, TAOC, ORAC) [123]. Twelve compounds were prepared through the reaction between selenosemicarbazones **191** and α-bromoketones **192** in a 1:1 mixture of EtOH/H_2_O as solvent (Scheme 52). All the compounds showed antioxidant activity in the DPPH and ORAC assays, with these being superior to ascorbic acid. Compounds without nitro substituents exhibited the highest antioxidant activity amongst those tested, with **193a** having the most potent. Compounds with nitro groups at the *ortho*- and *para*-positions displayed similar activities to the non-substituted and Me-derived ones in the DPPH test. However, in the ORAC test, compounds containing an *ortho*-nitro group were less active. Ultimately, the order of activities observed was **193a** > **193b** > **193c**. In the TAOC and TRP assays, the selenazolyl-hydrazones **193** did not yield significant results.

**Scheme 53:**
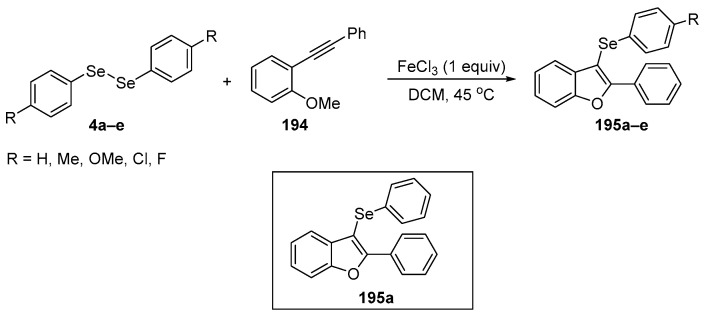
Synthesis of 2-phenyl-3-(arylselanyl)benzofurans **195**.

The antioxidant mechanisms involved in the antidepressant-like action of 2-phenyl-3-(arylselanyl)benzofuran **195a** were evaluated in vitro and in vivo by Bortolatto et al. [124]. Five compounds were prepared following the procedure of Zeni and co-workers [125] (Scheme 53). The intramolecular cyclization of *ortho*-alkynyl anisoles **194** with diaryl diselenides **4** in the presence of equivalent amount of FeCl_3_ afforded the expected benzofurans **195** selectively. Prior to in vivo tests, screening of the compounds **195a**–**e** was carried out to assess their ability to reduce lipid peroxidation (determined by TBARS) in brain tissue. Compound **195**, when present at ≥10 μM, demonstrated protective effects against reactive species generation, with **195a** giving the lowest IC_50_ and I_max_ values (IC_50_: 9.7 μM; I_max_: ~76%). In the TBARS test, **195a** displayed the highest effectiveness (IC_50_: 122 μM; I_max_: ~47%). Subsequently, the effects of treatment with **195a** at a dose of 50 mg/kg were evaluated in the hippocampus of male mice exposed to *p*-CPA (a serotonin depletion agent). Parameters evaluated included lipid peroxidation levels, total thiol content, CAT activity, and Na^+^/K^+^-ATPase activity. *p*-CPA exposure increased lipid peroxidation and CAT activity and reduced total thiol content and Na^+^/K^+^-ATPase activity, whereas treatment with **195a** reversed the decline in total thiols and inhibited lipid peroxidation in the brain tissue, consistent with potential neuroprotective effects.

## 4. Conclusions and Perspectives

The synthesis and testing of new organoselenium molecules for biological activities is a field of rapid development. In recent years, many new compounds have been prepared, with many of these being molecular hybrids of known bioactive compounds and organoselenium groups, or unprecedented molecules. In the search for the “Holy Grail” of organoselenium compounds, i.e., compounds that can mimic GPx, a diversity of synthetic approaches has been explored, and multiple bioassays have been used to assess bioactivity. For many years, Ebselen was the most likely candidate to be marketed. It has been tested in several clinical trials and is currently in a Phase 3 study to treat acute noise induced hearing loss, on the basis of its GPx-like activity and anti-inflammatory properties. As discussed above, dozens of studies have reported the synthesis of organoselenium compounds, with the biological activity evaluated mainly in vitro. This is not ideal, and an important limitation, as it is clear from the data described above that (in multiple cases) the in vivo activities differ from those observed in vitro. The in vitro data tend to be over-optimistic with regard to activity, as alternative reactions often occur in vivo, and therefore it is critical that in vivo testing is undertaken. Furthermore, a number of the assays commonly used for in vitro testing are artefact prone and non-physiological (e.g., the DDPH, ABTS, FRAP assays). Use of more complex reaction systems (e.g., tests on cultured or primary cells, insects, and worms, which are less costly than animals) are recommended before animal testing, as are the use of more precise and accurate assays of biological oxidation—particularly those that do not involve added agents, such as fluorescent dyes or probes that may be artefact prone or perturb the systems under study [12,18,126]. Animal studies also have limitations, and consideration needs to be given to dosing methods, pharmacokinetics, metabolism, and multiple other factors, including the possibility of long-term toxicity arising from bioaccumulation of either the parent compound or metabolites. In this respect, highly water-soluble compounds (cf. selenosugars that are rapidly excreted) and those that are resistant to metabolic biotransformation are probably going to be beneficial.

It is likely to be only a matter of time before positive compounds are identified, as new attractive and efficient protocols are emerging to make complex in vitro assays more accessible and reliable. In particular, a number of organic selenium compounds have shown promising protective effects through different mechanisms, including mimetic activities of antioxidant enzymes and modulation of antioxidant genes and redox signaling, which might be considered in the development of new therapeutic approaches for oxidative stress-related diseases.

Regarding the synthesis of organoselenium compounds, the future development in this field will be based on the green chemistry principles, including but not limited to using alternative energy sources, like ultrasound, light, and mechanochemistry, flow chemistry, green solvents, and atom-economic approaches. As demonstrated in this review, this has already been a trend, but there is still room to evolve in this area.

## Data Availability

No new data were created or analyzed in this study. Data sharing is not applicable to this article.
